# Loss of REST in breast cancer promotes tumor progression through estrogen sensitization, MMP24 and CEMIP overexpression

**DOI:** 10.1186/s12885-022-09280-2

**Published:** 2022-02-17

**Authors:** Ashley S. Cloud, Aditya M. Vargheese, Sumedha Gunewardena, Raeann M. Shimak, Sornakala Ganeshkumar, Easwari Kumaraswamy, Roy A. Jensen, Vargheese M. Chennathukuzhi

**Affiliations:** 1 Department of Molecular and Integrative Physiology, University of Kansas Medical Center, Kansas City, KS USA; 2 The University of Kansas Cancer Center, Kansas City, KS USA; 3 University of Kansas, Lawrence, KS USA; 4 Department of Biostatistics, University of Kansas Medical Center, Kansas City, KS USA; 5 Department of Pathology and Laboratory Medicine, University of Kansas Medical Center, Kansas City, KS USA; 6 Department of Cancer Biology, University of Kansas Medical Center, Kansas City, KS USA; 7 Department of Anatomy and Cell Biology, University of Kansas Medical Center, Kansas City, KS USA

**Keywords:** REST, CEMIP, MMP24, Breast cancer, Estrogen signaling, Tumor progression

## Abstract

**Background:**

Breast cancer is the most common malignancy in women, and is both pathologically and genetically heterogeneous, making early detection and treatment difficult. A subset of breast cancers express normal levels of *REST* (repressor element 1 silencing transcription factor) mRNA but lack functional REST protein. Loss of REST function is seen in ~ 20% of breast cancers and is associated with a more aggressive phenotype and poor prognosis. Despite the frequent loss of REST, little is known about the role of REST in the molecular pathogenesis of breast cancer.

**Methods:**

TCGA data was analyzed for the expression of REST target genes in breast cancer patient samples. We then utilized gene knockdown in MCF-7 cells in the presence or absence of steroid hormones estrogen and/ progesterone followed by RNA sequencing, as well as chromatin immunoprecipitation and PCR in an attempt to understand the tumor suppressor role of REST in breast cancer.

**Results:**

We show that REST directly regulates *CEMIP* (cell migration-inducing and hyaluronan-binding protein, KIAA1199) and *MMP24* (matrix metallopeptidase 24), genes known to have roles in invasion and metastasis. REST knockdown in breast cancer cells leads to significant upregulation of CEMIP and MMP24. In addition, we found REST binds to RE-1 sites (repressor element-1) within the genes and influences their transcription. Furthermore, we found that the estrogen receptor (ESR1) signaling pathway is activated in the absence of REST, regardless of hormone treatment.

**Conclusions:**

We demonstrate a critical role for the loss of REST in aggressive breast cancer pathogenesis and provide evidence for REST as an important diagnostic marker for personalized treatment plans.

**Supplementary Information:**

The online version contains supplementary material available at 10.1186/s12885-022-09280-2.

## Background

Breast cancer is the most common malignancy in women and is the second leading cause of cancer related deaths, after lung cancer [1]. Breast cancer is described as a heterogeneous disease consisting of clinically and pathologically diverse subtypes. Each subtype has a distinct behavior and a variable response to treatment making it difficult to determine the best therapy for each patient [2]. Additionally, multiple subtypes can exist within a tumor and subtypes can differ between the primary tumor and a metastatic site [3]. Progress has been made by using gene expression signature-based tools that can predict disease outcome and help form more effective treatment plans. This has led to a better understanding of the intracellular signaling pathways that are responsible for tumorigenesis and metastasis. Using these pathways, new targets for cancer therapy have been identified [4].

RE1-silencing transcription factor (REST), also known as Neuron-Restrictive Silencer Factor (NRSF), has been shown to have a unique gene signature when the protein function is lost [5]. REST is a transcriptional repressor that silences neuronal genes in the periphery [6]. REST binds to repressor element-1 (RE-1) sites found in the regulatory regions of its target genes. Its role is to act as a molecular scaffold and recruit co-repressors and chromatin remodelers to epigenetically silence target gene transcription [7]. REST binding differs based on the cell type and the differentiation state of the cell. This can alter the complexes present and the degree of gene repression [8]. However, REST has also been shown to be a tumor suppressor in mammary epithelial cells [9]. Loss of REST function, which occurs in 20% of breast cancers (RESTless), is associated with increased relapse and disease aggression. Additionally, RESTless tumors were associated with a unique gene signature that correlated with increased lymph node metastasis [5]. Previous studies have also demonstrated that downregulation of *REST* stimulated proliferation in MCF-7 cells and lead to upregulation of insulin receptor substrate 1 (IRS1) [10, 11]. Diseases including colon cancer, lung cancer, and uterine leiomyomas have also been associated with loss of REST function. These studies show upregulation of REST target genes lead to aberrant signaling and tumor pathogenesis [12–15]. However, a mechanism for how REST is associated with disease aggression or metastasis has yet to be elucidated. Additionally, in spite of finding putative REST gene signatures in breast cancers, direct transcriptional regulation of genes aberrantly expressed in RESTless breast cancers has not been investigated. Such studies are essential to correlate gene polymorphisms and alterations in epigenetic marks to the development of aggressive breast cancers.

A better understating of how loss of REST leads to increased tumor progression or aggressive clinical behavior requires an extensive look into REST target genes. Our research on the role of REST in uterine fibroids indicated that the loss of REST alters steroid hormone response in the uterus (unpublished data). In breast cancer, hypersensitivity to estrogen leads to estrogen induced cell proliferation, epithelial-mesenchymal transition (EMT), and cancer metastasis [16]. We utilized gene knockdown in MCF-7 cells in the presence or absence of estrogen and progesterone followed by RNA sequencing, as well as chromatin immunoprecipitation in an attempt to understand the tumor suppressor role of REST in breast cancer. Similar to what we saw in the uterus, estrogen signaling was activated upon *REST* knockdown in MCF-7 cells. Furthermore, two of the most significantly affected genes after the loss of REST were *CEMIP* (cell migration-inducing and hyaluronan-binding protein, KIAA1199) and *MMP24* (matrix metalloproteinase-24), which have been shown to be upregulated in many different cancers including breast, colorectal, gastric, and prostate cancer [17–21].

CEMIP was first identified as an inner ear specific protein and mutations in the *CEMIP* gene lead to non-syndromic hearing loss [22]. However, recent studies have shown increased CEMIP expression in cancers is associated with poor patient survival and leads to increased proliferation, EMT, invasion and migration [19, 23, 24]. CEMIP has been shown to promote brain metastasis, hyaluronan depolymerization in skin fibroblasts, and activation of Wnt/B-catenin signaling pathways [18, 25, 26]. Additionally, CEMIP affects EMT signaling molecules including TGF-β, PI3K, and AKT in a mouse xenograft NSCLC tumor model [25, 27]. Further studies are needed to determine how *CEMIP* is regulated, but reduced H3K27Me3 has been indicated to play a role in its activation [28]. Furthermore, MMP24 is a membrane type MMP (MT-MMP) and is expressed on the surface of the cell where it mediates cleavage of extracellular matrix (ECM) proteins. MMP24 known substrates include CD44, cadherin 2, and MMP2. MMP24 promotes neuronal migration, contributes to Alzheimer’s pathogenesis and is necessary for lung cancer invasion and metastasis [29–32]. Studies in breast cancer, endometriosis, and lung cancer have all shown *MMP24* overexpression [20, 21, 30, 33]. Unfortunately, little is known about the role of MMP24 in breast cancer and what factors contribute to its regulation. Therefore, further studies are needed to clarify how CEMIP and MMP24 are regulated in breast cancer.

In this study, we show that REST target genes are dysregulated across different cancers and breast cancer subtypes. Additionally, we found that REST directly regulates genes playing key roles in disease recurrence, invasion, and metastasis, including *CEMIP* and *MMP24*. When REST is lost in breast cancer these genes become aberrantly expressed. Furthermore, we demonstrate that loss of REST in MCF-7 cells activates the estrogen receptor signaling pathway, regardless of the presence of estrogen. This activation leads to upregulation of REST and estrogen receptor alpha (ERα) target genes that play a role in cell proliferation and migration. Identifying these gene pathways that are specific to RESTless tumors could help in formulating personalized treatment modalities in the future. Our results show loss of REST may contribute to an aggressive phenotype by upregulating invasive and metastatic genes and altering steroid hormone signaling pathways in breast cancer.

## Methods

### Data mining

TCGA (The Cancer Genome Atlas) data was analyzed for the expression of REST target genes in many different types of cancer, including breast cancer patient samples, using the Broad Institute FireBrowse portal (http://firebrowse.org/). REST downstream gene expression levels in breast cancer sub-types were investigated in the following GEO series (https://www.ncbi.nlm.nih.gov/geo/), GSE26304 (normal, DCIS and IDC patients), GSE2034 (ER + and ER- patients), and GSE19615 (TNBC and non-TNBC patients) [34–36]. Sixteen ChIP-seq datasets (Additional file 1) from ENCODE [37] were used to determine potential REST target genes by examining REST binding to RE1 sites located within or near genes of interest. REST DNA binding sites were identified by the presence of both a strong ChIP-peak and REST consensus sequence within the peak region with at least 80% identity.

### Cell Culture

MCF-7 cells were obtained from ATCC (Manassas, VA) and authenticated by STR DNA Fingerprinting at MD Anderson. Cells were cultured in DMEM (Corning, Tewksbury, MA) containing 10% FBS (R&D Systems, Inc., Minneapolis, MN) and 1% penicillin/streptomycin (Gibco, Waltham, MA) in a humidified 5% CO_2_ atmosphere at 37 °C. MDA-MB-231 cells were obtained from ATCC (Manassas, VA), and authenticated by STR DNA Fingerprinting at Arizona Research labs. They were cultured in Leibovitz’s L-15 medium (Corning, Tewksbury, MA) containing 10% FBS (R&D Systems, Inc., Minneapolis, MN) in a humidified incubator at 37 °C. HCC1937, MDA-MB-468, and T-47D cell lines were obtained from ATCC (Manassas, VA) and HCC1937/*WT BRCA1 (*hereafter referred to as HCC1937 +) were obtained from Dr. Jensen [38]. Cell culture conditions have been previously described [38]. Cell lines were tested for mycoplasma using the ATCC Universal Mycoplasma Detection kit (Manassas, VA).

### Transfections

Cultured MCF-7 cells were plated in 6-well plates at 300,000 cells per well in antibiotic free media. After 24 h at 37 °C, 5%CO_2_, cells were transfected with a 100 pmol mixture of Silencer Select siRNAs (S11934, S11932, S119323) to *REST* from Ambion (Life Technologies, Carlsbad, CA) using Lipofectamine 2000 transfection reagent (Invitrogen, Carlsbad, CA) according to manufacturer’s protocol. Control experiments included ON-TARGETplus nontargeting scrambled siRNA 2 (ThermoFisher Scientific, Waltham, MA). After 24 h, transfected cells were treated with vehicle (PBS), estradiol (10 nM), progesterone (1uM), or estradiol and progesterone in charcoal stripped serum, phenol red free medium (Lonza, Morristown, NJ). After 24 h of treatment, RNA and protein extracts were collected and analyzed.

For RT-qPCR and western blot validation of RNA sequencing results, cultured MDA-MB-231 and MCF-7 cells were plated at 500,000 cells per well in antibiotic free media. After 24 h, cells were transfected with Silencer Select siRNAs (S11934, S11932, S119323) to *REST* from Ambion (Life Technologies, Carlsbad, CA) using Lipofectamine 2000 transfection reagent (Invitrogen, Carlsbad, CA) according to manufacturer’s protocol. Control experiments included ON-TARGETplus nontargeting scrambled siRNA 2 (ThermoFisher Scientific, Waltham, MA). After 24 h, transfected cells were collected for chromatin, protein, and RNA extracts, and after 48 h transfected cells were collected for protein and RNA.

### RNA sequencing and functional and pathway analysis

Total RNA was isolated from MCF-7 cells using TRIzol Reagent (Invitrogen, Carlsbad, CA) according to manufacturer’s protocol. After quality control, RNA samples were sequenced by the Genome Sequencing Facility at the University of Kansas Medical Center using the Illumina NovaSeq 6000 sequencing machine (Illumina, San Diego, CA). Strand specific 100 cycle paired end reads were generated at around 41.5 to 43.5 million reads per sample of which around 99% mapped to the reference genome giving 39 to 41 million uniquely mapped reads per sample. The read quality was assessed using the FastQC software [39]. On average, the per sequence quality score measured in the Phred quality scale was above 30 for all the samples. The reads were mapped to the human genome (GRCh38/hg38) using STAR software [40]. Transcript abundance estimates were calculated using the RSEM software [41]. Expression normalization and differential gene expression calculations were performed in edgeR to identify statistically significant differentially expressed genes [42]. A false discovery rate (FDR) was calculated for the significantly differentially expressed genes using the Benjamini and Hochberg procedure [43]. A FDR cutoff of 0.05 or less with an absolute fold change difference greater than or equal to 1.5 was used as the cutoff criteria for selecting significantly differentially expressed genes. Biological, functional, and pathway analysis were performed using Ingenuity Systems Pathway Analysis software (IPA, QIAGEN, Germantown, MD) on the significantly differentially expressed genes between MCF-7 cells with siREST and MCF-7 cells with scrambled siRNA as control. Data deposited in the NCBI Gene Expression Omnibus (GEO, GSE173857).

### Gene Expression Omnibus (GEO) data analysis

Microarray data downloaded from the GEO database were background corrected, normalized and gene-level summarized using the Robust Multichip Average (RMA) procedure. The normalized log expression data were used for downstream differential expression and clustering analysis. Differential expression analysis was performed using the Partek Genomic suite (v 6.5, Partek Inc., St. Louis, MO). *P*-values were adjusted (FDR) for multiple hypothesis testing by the Benjamini and Hochberg procedure [43]. Genes with multiple transcripts in the microarray were resolved by taking the transcript with maximum variance across samples. Genes with differential expression statistics between any two conditions in the dataset passing the cutoff criteria of absolute fold difference ≥ 1.5 and FDR ≤ 0.05 were considered significant for further analysis. Prior to clustering, gene expression data were standardized to the geometric average of the three housekeeping genes MRPL19, PSMC4, and PUM1 by subtraction. Only REST target genes and breast cancer genes downstream of them were used for clustering. Independent hierarchically clustering was performed on the Euclidian pair wise distance of the row standardized gene expression values linked using the Ward’s method. All clustering computations were performed in Matlab (R2018b, The MathWorks Inc, Natick, MA).

### Protein extraction and western blotting

Cells were lysed in 1 × cell lysis buffer (Cell Signaling Technology, Davers, MA) supplemented with protease and phosphatase inhibitor cocktails (Sigma-Aldrich Co., LLC, St Louis, MO). Samples were sonicated and centrifuged at 18,000xg at 4 °C. Protein was quantified using Bio-Rad Protein Assay (Bio-Rad, Hercules, CA). Western blots were performed as described previously [14]. Proteins were detected using SuperSignal West Pico PLUS Chemiluminescent Substrate (ThermoFisher Scientific, Waltham, MA) and blots were imaged using BioRad ChemiDoc MP (BioRad, Hercules, CA). The following antibodies were used: REST (Millipore, #07–579), Beta-actin (Genescript, #A01865), CEMIP (Novus Biologicals, #45,750,002) and anti-rabbit-HRP (Promega). For REST and CEMIP quantification, ImageJ software was used to measure band intensities and normalized to B-actin. Original western blot images can be found in Additional file 11.

### RNA isolation and RT-qPCR analyses

Total RNA was isolated from cells using TRIzol Reagent (Invitrogen, Carlsbad, CA) according to manufacturer’s protocol. After quantitation using Nanodrop spectrophotometer, aliquots of RNA were reverse transcribed using the High-Capacity cDNA Reverse Transcription kit (Applied Biosystems, Foster City, CA). Using the Applied Biosystems QuantStudio 7 Flex real-time PCR system and TaqMan Universal PCR Master Mix (Applied Biosystems, Foster City, CA), TaqMan assays (Additional file 2) were used to quantify gene expression using the delta delta C(T) method in comparison with 18 s rRNA [44].

### Semi-quantitative ChIP-PCR

ChIP assays of cultured MCF-7 were performed using the SimpleChIP Plus Enzymatic Chromatin IP Kit (Cell Signaling Technology, Davers, MA). Anti-REST ChIPAb + antibody (Millipore, #17–641), Anti-Histone H3 (Cell Signaling Technology, #4620), RNA Polymerase II (Millipore, #05–623), H3K27Me3 (Millipore, #07–449), and Rabbit IgG (Cell Signaling Technology, negative control) were used according to manufacturer’s protocol. PCR primers spanning the conserved RE1 elements in *MMP24* and *CEMIP* were designed based on the genomic sequence. The primer sequences are found in Additional file 3. For quantification, ImageJ software was used to measure band intensities and the samples were normalized to their respective inputs. Original gel images are included in Additional file 11.

### Statistical analyses

Quantitative experiments including RT-qPCR and densitometry of western blots and semi-quantitative ChIP-PCR, were repeated with three independent biological replicates. Statistical significance was determined using student’s T-test to determine change from control sample. Samples were normalized to their respective inputs or to actin. From RNA-sequencing results, genes used in Ingenuity pathway Analysis were filtered for *FDR* < 0.05 and an absolute fold change of 1.5 or higher.

## Results

### Aberrant expression of REST target genes across different cancers

**Fig. 1:**
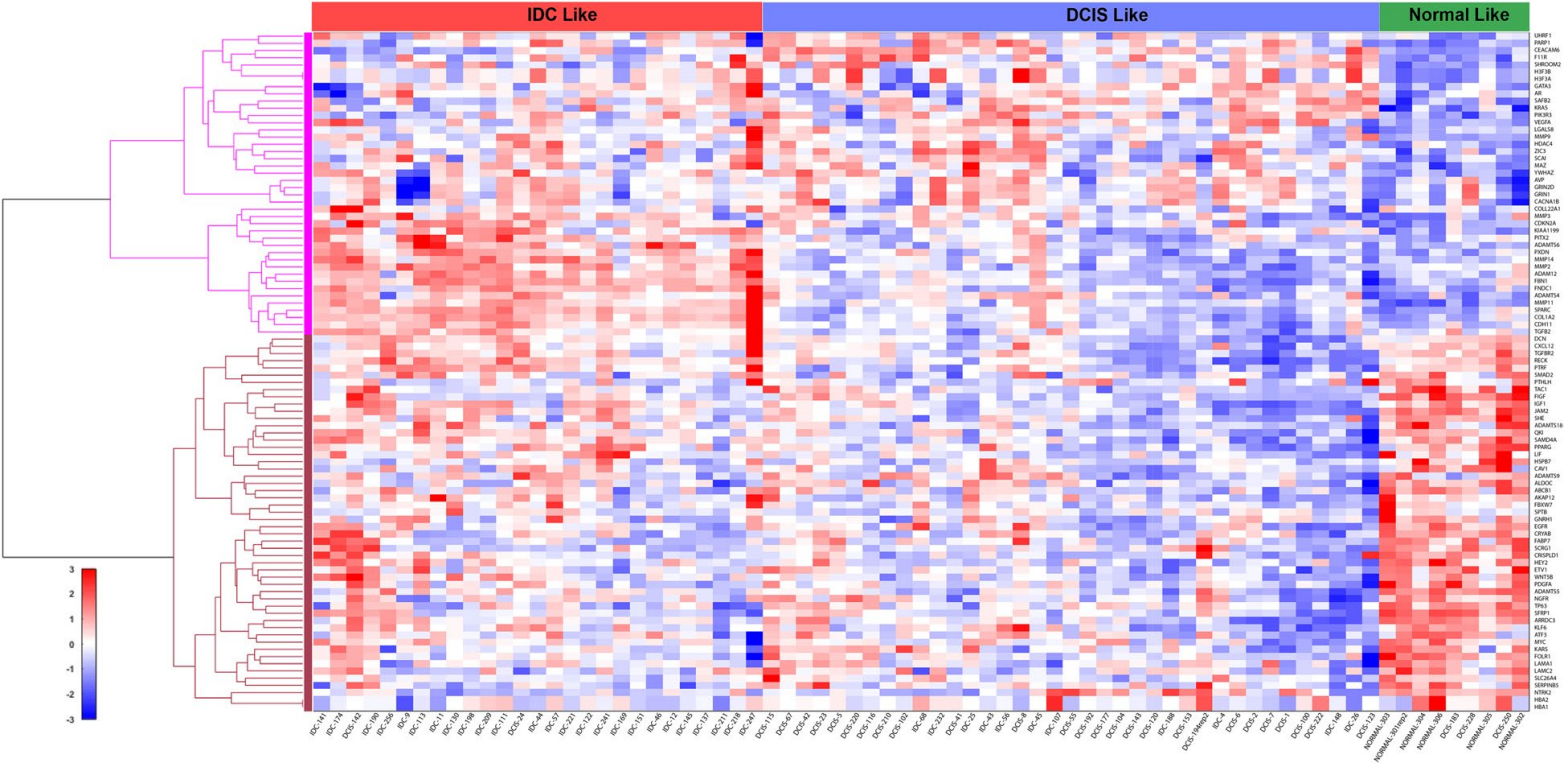
Analysis of REST target genes in cancer. Hierarchical clustering and gene expression heatmap of GSE26304 data analysis of REST targets’ and downstream gene expression profiles in breast cancer types, normal like, invasive ductal carcinoma (IDC), and ductal carcinoma in situ (DCIS)

In an effort to identify cancers in which REST targets were dysregulated, we analyzed gene expression profiling data in TCGA using FireBrowse. Analysis of the various cancers revealed that several putative REST target genes, including Polo Like Kinase 1 (PLK1), ADAM Metallopeptidase Domain 12 (ADAM12), Troponin T1 (TNNT1), and cell migration-inducing and hyaluronan-binding protein (CEMIP, KIAA1199) were highly dysregulated (Additional file 4). Since REST plays a crucial role in the pathogenesis of uterine fibroids [14] and its steroid hormone response (unpublished data), we wanted to investigate if similar mechanisms of steroid hormone sensitivity and tumor pathogenesis exist in breast cancer. When broken down into breast cancer subtypes based on invasiveness including normal like, invasive ductal carcinoma (IDC), and ductal carcinoma in situ (DCIS) we see differential expression of a novel set of REST target genes across all the subtypes (Fig. 1). Additionally, we found the more invasive and aggressive subtype, IDC, has higher REST target gene expression. These observations were similar to those seen by Wagoner et al., where loss of REST function is associated with a unique gene signature [5]. Analysis of REST target gene expression in the presence and absence of hormone receptors showed significant differential regulation of a subset of REST target genes (Additional file 5). This distinct regulation was also seen when we compared triple negative breast cancer (TNBC) to non-TNBC (Additional file 5). The subset of REST target genes that are upregulated in ER- and TNBC include VEGFa, CDKN2a, SFRP1, EGFR, MYC, LIF, MMP9, and KIAA1199 (CEMIP). These genes play important roles in breast cancer progression. CEMIP has recently been shown to contribute to metastasis and invasion in a number of different cancers [23, 24]. Unfortunately, the regulation of these genes are not well understood. In ER + and non-TNBC we see ADAM12, COL1A2, IGF1, AR, MMP2, CAV1, SMAD2, and GATA3 are upregulated. Many of these genes contribute to multiple cancer associated pathways [20, 45, 46]. REST’s regulation of these genes is important to consider as RESTless tumors will behave differently in ER + vs ER- subtypes. This difference could affect RESTless tumors response to hormone-based therapies. Using a RESTless gene signature could allow for hormone-based treatment options to be better tailored to RESTless tumors compared to tumors that contain REST. In order to further investigate loss of REST in breast cancer we analyzed REST protein levels in different breast cancer cell lines. MCF-7 cells were used to represent hormone responsive breast cancers while MDA-MB-231 cells represent TNBC. REST expression was found in both MCF-7 and MDA-MB-231 with MCF-7 cells expressing higher levels of endogenous REST. Additionally, REST was effectively knocked down in both cell lines using siRNAs targeting *REST* (Additional file 6).

### Loss of REST leads to activation of ERα signaling

**Fig. 2:**
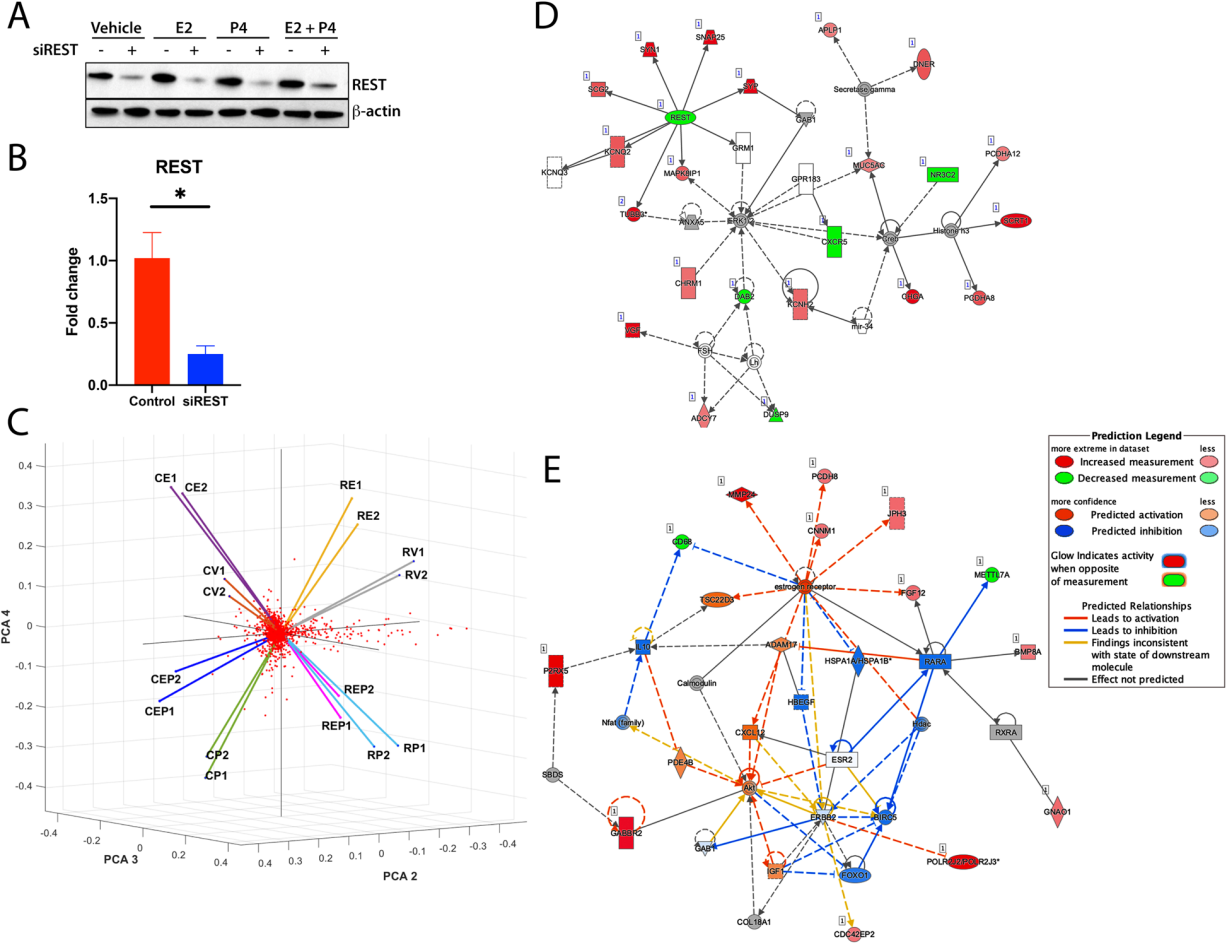
Loss of REST leads to activation of ER⍺ signaling. **A** Downregulation of REST upon siRNA transfection. MCF-7 cells were transfected with control siRNA or siRNA directed to REST. Twenty-four hours after transfection, cells were treated with vehicle (PBS), estradiol (E2), progesterone (P4) or both. RNA-sequencing was performed with technical replicates (*n* = 2). Forty-eight hours after transfection REST protein levels were assayed by immunoblotting. Blot has been cropped for clarity. B-actin was used as a loading control. **B** REST mRNA level in MCF-7 cells upon REST siRNA transfection in vehicle treated cells (*n* = 3) (Representative image for siREST in all treated groups). **C** Principal component analysis (PCA) plot indicates distinct gene expression patterns based on treatment. Gene expression induced by *REST* knock down is asserted along PCA-2. Estradiol induced gene expression is asserted along PCA-3. Progesterone induced gene expression is asserted along PCA-4 (not in figure). **D** IPA identified REST downstream target genes in a network that are upregulated upon REST siRNA transfection. **E** Estrogen receptor signaling was shown to be upregulated in the REST siRNA, vehicle treated cells compared to the control. Upregulated genes shown in red, downregulated genes shown in green, predicted activation shown in orange, and predicted inhibition shown in blue. Error bars indicate ± SD, Student’s T-test was performed, *P < 0.05

We studied the regulation of gene expression in MCF-7 cells to investigate how loss of REST affected tumorigenic cell signaling pathways. First, *REST* was knocked down in cultured cells using specific siRNAs and 24 h post siRNA transfection, the cells were treated with vehicle (PBS), estradiol (10 nM), progesterone (1uM), or both. Comparing with siControl, the siRNAs targeting *REST* were efficient at significantly downregulating REST (*p* < 0.05) at the protein level and mRNA level (Fig. 2A and B). Aliquots of RNA from the samples were subjected to RNA sequencing in order to determine gene expression changes that are influenced by the loss of REST (GSE173857). *REST* knock down, treatment with estradiol and/or progesterone created distinct changes in the gene expression profile of the effected samples as seen by the principal component projection (PCA) of their expression (Fig. 2C). An agglomerative hierarchical clustering of the significantly differentially expressed genes between different treatment groups illustrates the distinct subsets of genes expressed under these conditions (Additional file 7). Ingenuity Pathway Analysis (Qiagen, IPA) was used to further understand tumorigenic pathways affected by the loss of REST. Table 1 shows the top fifteen genes that were significantly upregulated upon *REST* knockdown in the absence of hormonal treatment. Additional file 7 shows all these genes lie in Sub cluster 1. Table 2 and Additional file 7 (Sub cluster 4) show a large proportion of these genes are significantly altered by estradiol treatment. IPA predicted that the REST pathway was significantly inhibited (activation z-score -2.415, *p*-value 5.76E-09) in all of the MCF-7 siRNA treated groups (Fig. 2D). REST downregulation, indicated in green, leads to its target genes being overexpressed (red) (Fig. 2D). Additionally, IPA predicted that estrogen receptor signaling was significantly activated (activation z-score 2.433, *p*-value 0.0176) after *REST* knockdown even in the vehicle treated group (Fig. 2E). Investigation of the genes affected showed a large number of them are known to play a role in breast cancer (Additional file 8). Additionally, a highly significant increase in the number of genes upregulated by estrogen was observed in MCF-7 cells that lost REST expression (Additional file 8). These data suggest that knockdown of REST alters the cells’ response to sex steroid hormones, a fundamental aspect of breast cancer pathophysiology and may impact a patient’s response to available treatments.

**Table 1:** Genes that were upregulated after MCF-7 cells were transfected with REST siRNA and treated with vehicle. The top fifteen most significantly affected genes are represented in the table

Gene Name	Fold Change	FDR
SEZ6	449.2	9.6E-23
CPLX2	414.8	9.8E-81
KCNH6	316.9	6.3E-14
AMER3	289.4	2.9E-12
SCRT1	85.6	2.9E-83
GPR6	84.2	2.2E-03
RAB39A	46.8	7.1E-16
KCNC2	46.1	2.0E-22
CHRNB2	45.0	4.7E-97
UNC13A	39.0	4.9E-149
MMP24	20.3	1.0E-82
KCNK3	19.4	3.3E-38
RUNDC3A	18.6	2.1E-98
XKR7	18.6	2.4E-107
LRTM2	17.8	1.2E-16

**Table 2:** Genes that were upregulated after MCF-7 cells were transfected with REST siRNA and treated with estradiol. The top fifteen most significantly affected genes are represented in the table

Gene Name	Fold Change	FDR
CPLX2	328.3	8.92E-69
SCRT1	132.3	1.90E-100
UNC13A	90.1	4.09E-175
KCNC2	72.4	1.67E-21
PALM2-AKAP2	66.7	0.291744885
KCNK3	65.0	7.04E-39
KCNH6	56.7	2.79E-11
SEZ6	55.8	1.01E-43
RAB39A	53.7	1.84E-17
CHRNB2	46.1	6.13E-101
GPR6	28.2	0.001069982
AMER3	22.8	4.82E-07
RUNDC3A	22.7	3.13E-105
MMP24	19.6	4.64E-90
XKR7	19.3	5.55E-117

### Loss of REST increases target gene expression, including CEMIP and MMP24

**Fig. 3:**
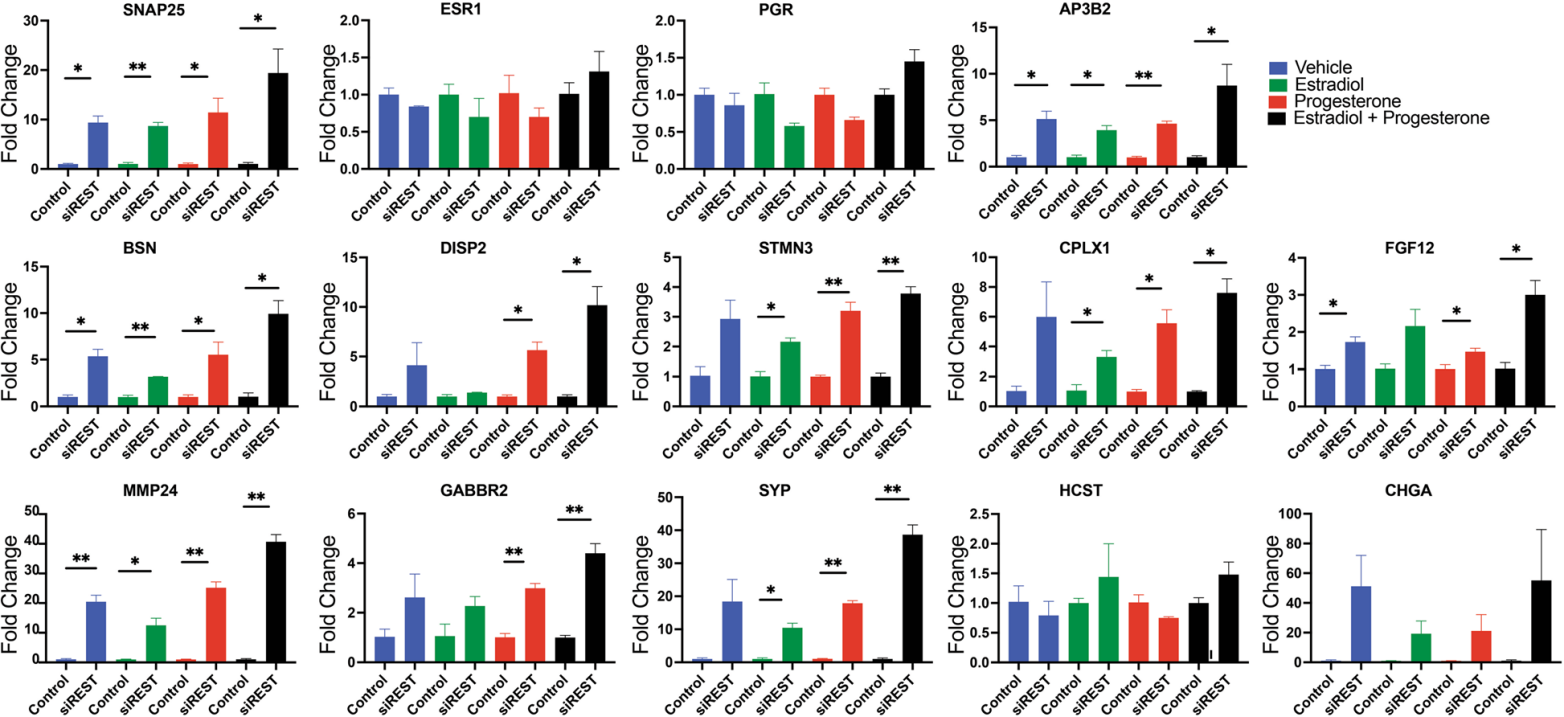
Gene expression analysis of REST knockdown in MCF-7 cells. Gene expression analysis using RT-qPCR showing siRNA knockdown of REST in MCF-7 cells treated with control (PBS), estradiol (10 nM), progesterone (1uM), or both leads to differences in overexpression of REST targets. Error bars indicate ± SD. Student’s t-test was performed, *P < 0.05,**P < 0.01

Analysis of RNAseq data from MCF-7 cells using IPA revealed that many of the aberrantly expressed REST target genes could contribute to disease progression. We prioritized a smaller number of genes that may be playing a role in this process. We selected genes that were known as ‘REST targets’ and novel genes that were potential REST targets based on available ChIP sequencing data sets (Additional file 1). We performed RT-qPCR using the siREST transfected MCF-7 samples. Expression of the steroid hormone receptors, estrogen receptor (*ESR1*) and progesterone receptor (*PGR*), remained stable when comparing control and *REST* knockdown samples. Known REST target genes including, *AP3B2*, *CPLX1*, *CHGA*, and *DISP2* all show upregulation in the knockdown samples (Fig. 3 and Additional file 7 Sub cluster 1). This result confirms a sufficient knockdown was achieved in the MCF-7 cells. Genes downstream of ERα signaling that were upregulated included *MMP24*, *GABBR2*, *SYP*, *STMN3*, *SNAP25*, *FGF12* and *BSN* (Fig. 3 and Additional file 7 Sub cluster 1). Upregulation of these genes changed depending on the hormone treatment present indicating variable responses to hormones could occur in the absence of REST.

**Fig. 4:**
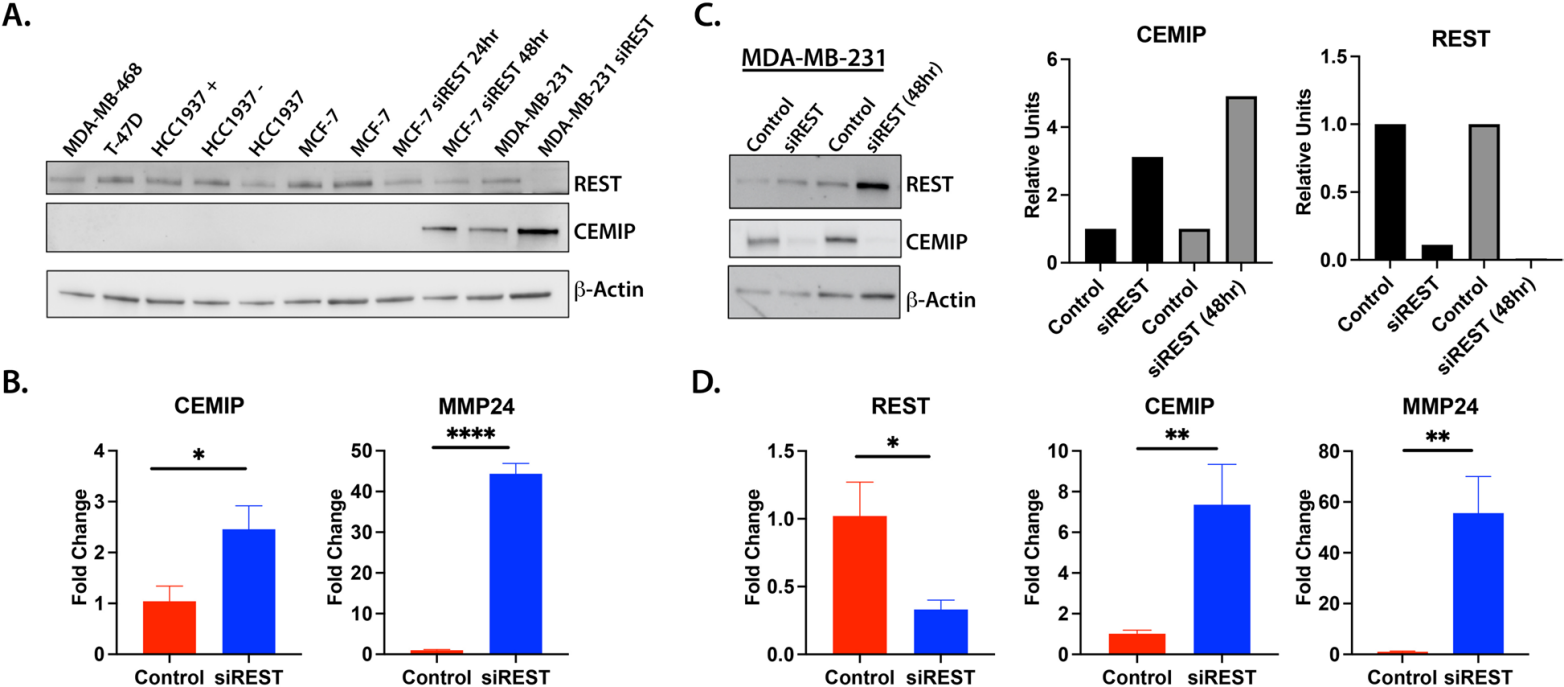
Breast cancer cell lines showing REST and REST target gene expression. **A** Western blot of REST and CEMIP expression in breast cancer cell lines MDA-MB-468, T-47D, HCC1937 + , HCC1937 -, HCC1937, MCF-7, MCF-7 with REST knockdown, MDA-MB-231, and MDA-MB-231 with REST knockdown. Beta-actin was used as a loading control. **B** *REST* knockdown using siRNA leads to a significant upregulation in *CEMIP* and *MMP24* expression in MCF-7 cells. **C** Western blot showing overexpression of CEMIP at 24 h and 48 h and decreased REST expression upon *REST* knockdown using siRNA. Densities for CEMIP and REST were calculated using ImageJ and normalized to beta-actin. **D** qPCR data showing a significant reduction in *REST* expression for MDA-MB-231 cells. *CEMIP* and *MMP24* were significantly upregulated in MDA-MB-231 cells treated with siRNA targeting *REST*. Western blots have been cropped for clarity. Error bars indicate ± SD, Unpaired T-test was performed, *P < 0.05, **P < 0.01

Additionally, IPA predicted genes involved in cell migration, cell–cell adhesion, and extracellular matrix organization including *CEMIP* and *MMP24* were significantly upregulated upon *REST* knockdown. CEMIP and MMP24 have been previously shown to be upregulated in breast cancer patient samples and cell lines [20, 47, 48]. However, transcriptional regulation of these genes in breast cancer is poorly understood. We further investigated how REST regulates the expression of these two genes with reported roles in breast cancer metastasis. First, we looked at the relationship of REST and CEMIP expression in different breast cancer cell lines, including MDA-MB-468, T-47D, HCC1937 + , HCC1937-, HCC1937, MCF-7, and MDA-MB-231. All cell lines showed high REST expression and no expression of CEMIP except for MDA-MB-231 (Fig. 4A). Here low REST expression was observed with higher levels of CEMIP. Upon REST knockdown, using siRNA, CEMIP expression was increased in MCF-7 and in MDA-MB-231 (Fig. 4A). We picked high REST expressing MCF-7 cells and low REST expressing MDA-MB-231 cells to further characterize REST’s regulation of *CEMIP* and *MMP24*. Using siRNAs specific to *REST*, we confirmed CEMIP was significantly upregulated upon REST knockdown with a 2.46 fold change (*p* = 0.011). Additionally, MMP24 was significantly upregulated (*p* = 0.0001) with a fold change of 44.6 upon REST knockdown (Fig. 4B). These results were also observed in RNA-sequencing data (CEMIP, FC = 1.58, FDR = 0.06; MMP24, FC = 20.33, FDR = 1.04E-82). REST knockdown was repeated in MDA-MB-231 cells with a 40% decrease in REST expression (*p* = 0.01). CEMIP showed a fold change of 7.35 (p = 0.0053) and MMP24 showed a fold change of 55.2 (*p* = 0.0027). Moreover, MDA-MB-231 had higher expression levels and CEMIP expression increased when the knockdown was extended to 48 h (Fig. 4C and D). MDA-MB-231 cells already express lower levels of REST protein compared to MCF-7 cells and this may contribute to the higher target gene expression levels (Fig. 4A and Additional file 6).

### REST binds to the RE1 sites within *CEMIP* and *MMP24*

**Fig. 5:**
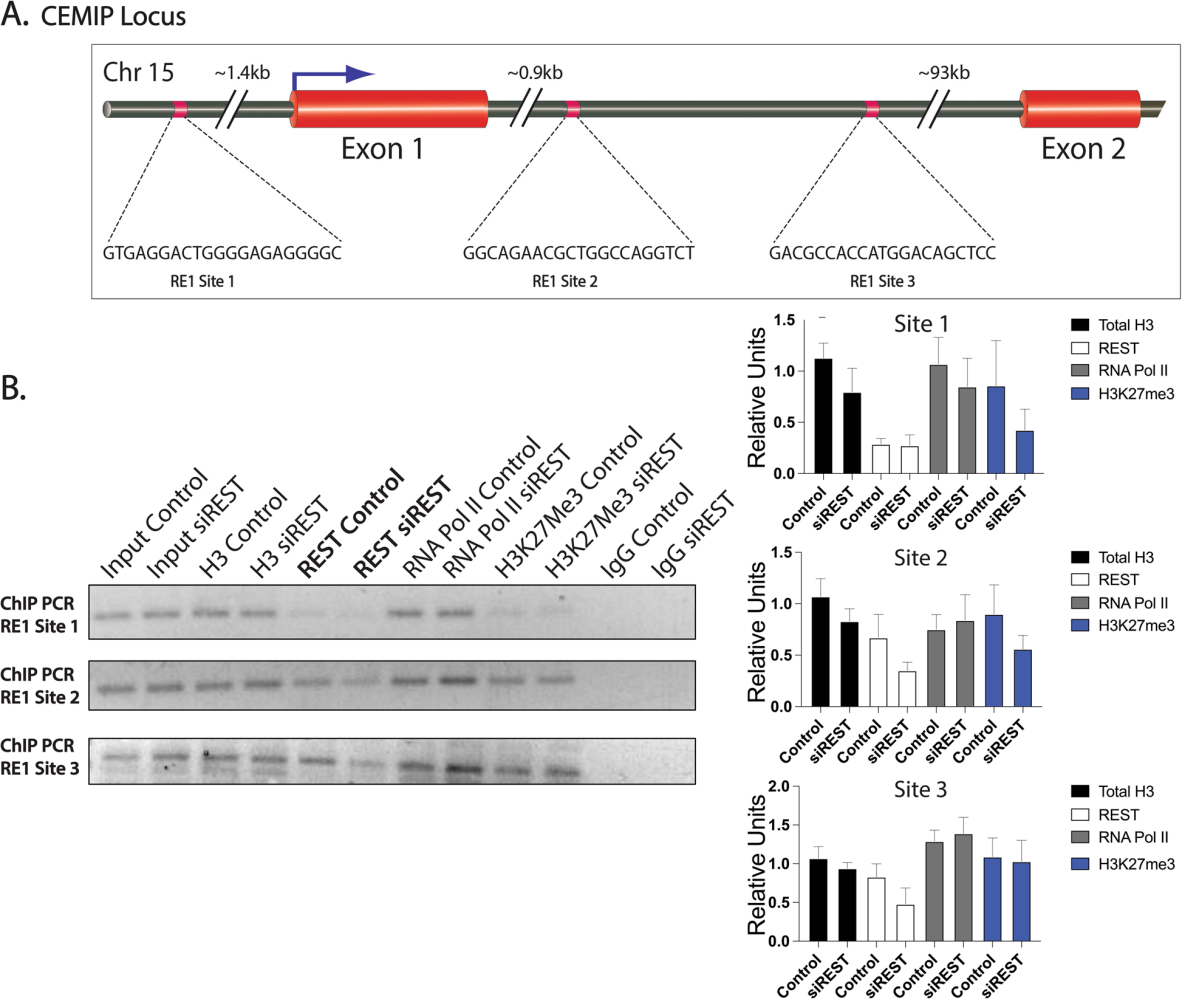
Semi-quantitative ChIP-PCR analysis of RE1 sites located within the CEMIP gene. **A** CEMIP gene locus showing REST binding sites (RE1 sites) and their locations relative to the transcription start site (TSS, + 1). **B** DNA immunoprecipitation analysis in MCF-7 cells (control vs siREST). ChIP using total H3 (positive control), REST, RNA polymerase II, H3K27Me3, and rabbit IgG (negative control) antibodies at RE1 Site 1, Site 2 and Site 3. Gels were cropped for clarity. A representative experiment of three independent biological replicates is shown. Relative densities (*n* = 3) were quantified in ImageJ and normalized to the respective inputs. Error bars indicate ± SD

Based on our findings that loss of REST and high CEMIP levels are positively correlated, we next wanted to see if REST binds within the CEMIP gene located on chromosome 15. REST binding sites, known as RE1 sites, were found in several locations of the CEMIP gene (Additional file 9). Site 1 (-1501) is located upstream of the transcription start site (+ 1) in the promoter region. Site 2 is located in intron 1 (+ 1022) and site 3 is located at + 1442 within the first intron (Fig. 5A). Interestingly, site 2 falls within a CpG island in the first intron of CEMIP. Kuscu et al. showed this CpG island (+ 525 to + 1059) is methylated in nonaggressive or minimally expressing CEMIP cell lines, including MCF-7. However, in aggressive breast cancer cell lines, MDA-MB-231, this CpG island is shown to be demethylated [49]. Given the role of REST in the epigenetic regulation of a multitude of its target genes, we sought to confirm the association of REST with its binding consensus sequence using semi-quantitative ChIP-PCR analysis in MCF-7 cells transfected with siControl or siREST. Cross-linked chromatin from the cells was immunoprecipitated using anti-Histone H3 (positive control), anti-REST, anti- RNA polymerase II, anti-H3K27Me3, and anti-IgG (control) antibodies. Three primer pairs for *CEMIP* were designed for semi-quantitative PCR as seen in Additional file 3. ChIP- PCR results indicated that REST was not associated with the RE1 sequence site 1 (-1501) in the control or in siREST knockdown MCF-7 cells (Fig. 5B). Site 2 (+ 1022) and site 3 (+ 1442) showed REST binding to the RE1 sequences in the control non-targeting siRNA treated samples (Fig. 5B). Additionally, the enrichment of these two RE1 sites decreased in the chromatin immunoprecipitants after REST knockdown (Fig. 5B), indicating that the interaction was REST specific. Additionally, REST knockdown appears to change binding of RNA polymerase II and H3K27Me3 in the siREST samples (Fig. 5B). However, future experiments using ChIP qPCR are required to confirm these epigenetic/ transcriptional changes upon REST knockdown as well as in patient samples with loss of REST expression. Taken together, these data confirm REST binding within the CEMIP gene and loss of REST binding upon siREST knockdown. Our finding that REST associated sequence elements overlap with the CpG island within the CEMIP locus will have important implications on the epigenetic regulation of this gene by REST and in tumor metastasis.

**Fig. 6:**
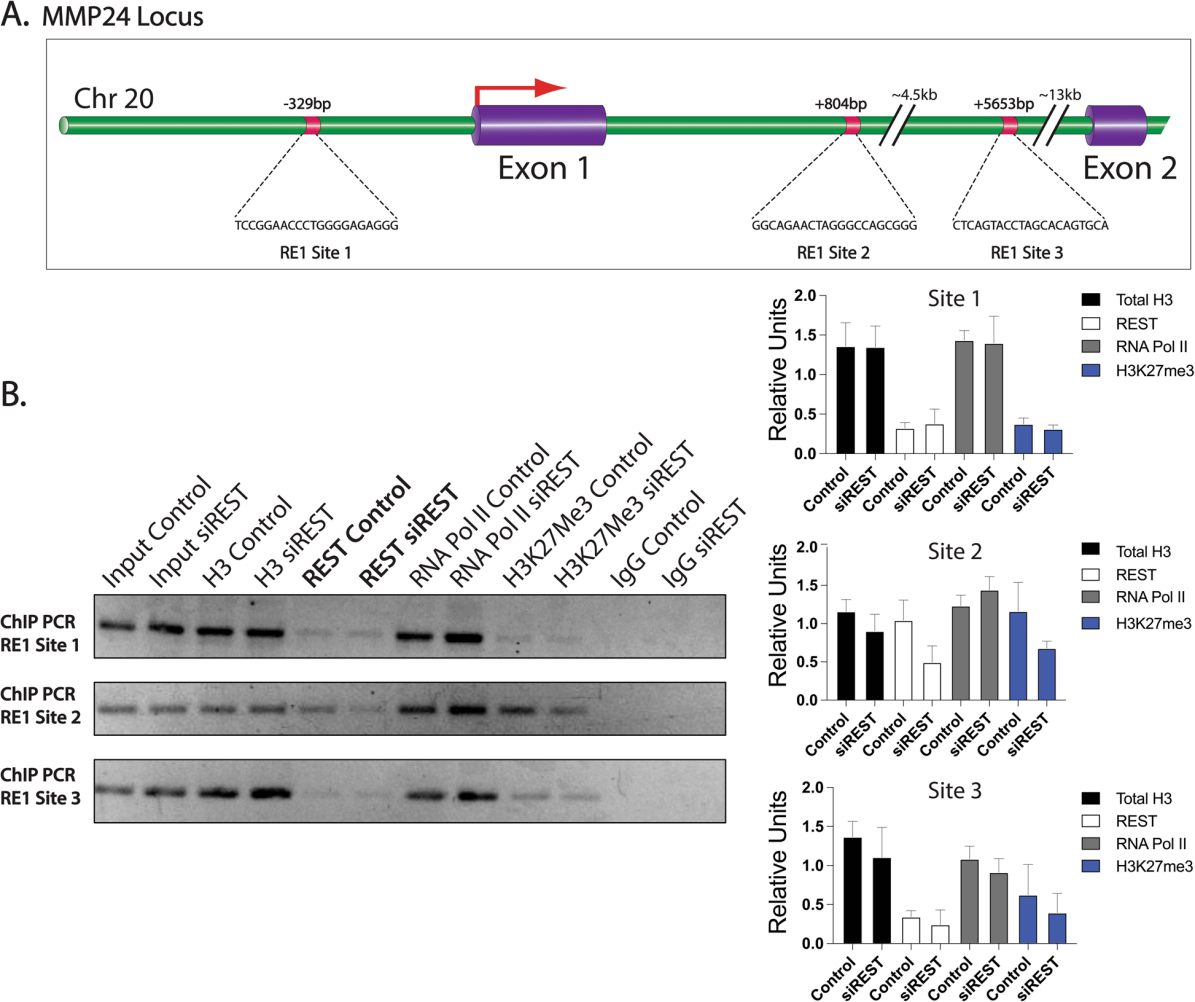
Semi-quantitative ChIP-PCR analysis of RE1 sites located with the MMP24 gene. **A** MMP24 gene locus showing REST binding sites (RE1 sites) and their locations relative to the transcription start site (TSS, + 1). **B** DNA immunoprecipitation analysis in MCF-7 cells (control vs siREST). ChIP using total H3 (positive control), REST, RNA polymerase II, H3K27Me3, and rabbit IgG (negative control) antibodies at RE1 Site 1, Site 2 and Site 3. A representative experiment of three independent biological replicates is shown. Gels were cropped for clarity. Relative densities (*n* = 3) were quantified in ImageJ and normalized to the respective inputs. Error bars indicate ± SD

While the loss of REST has previously been shown to increase *MMP24* expression in breast cancer cells, transcriptional regulation of MMP24 by REST has not been studied so far. We wanted to confirm that REST directly regulates MMP24 in breast cancer cells. Sequence analysis showed several putative RE1 sites in the MMP24 gene locus on chromosome 20 (Additional file 10). Site 1 (-329) is located in the promoter region of MMP24 while site 2 (+ 804) and site 3 (+ 5653) are located within the first intron (Fig. 6A). To confirm the association of REST with its binding consensus sequence, we performed semi-quantitative ChIP-PCR analysis in MCF-7 cells transfected with siControl or siREST. Cross-linked chromatin from the cells was immunoprecipitated using anti-histone H3 (positive control), anti-REST, anti-RNA polymerase II, anti-H3K27Me3, and anti-IgG (control) antibodies. Three primer pairs for *MMP24* were designed for semi-quantitative PCR as seen in Additional file 3. RE1 site 1 (-329) and site 3 (+ 5653) were not bound by REST. Site 2 showed REST was bound and this interaction was decreased by the REST knockdown (Fig. 6B). These results suggest that the loss of REST may alter the epigenetic regulation of MMP24, a matrix metalloproteinase with putative roles in breast cancer progression and metastasis.

## Discussion

In an effort to understand the mediators of aggressive tumor growth, invasion and metastasis in RESTless cancers, we analyzed available gene expression datasets for REST target gene signatures. We found REST target genes are frequently dysregulated in different types of cancers, including breast cancer, without a concurrent loss of *REST* mRNA expression (Fig. 1 and Additional file 4). This is not unexpected, given the frequent loss of REST protein by ubiquitin mediated proteolysis in various cancers [8]. REST is known to be regulated at the post transcriptional level without altering its mRNA levels [50]. Ubiquitinoylation and proteasomal degradation by beta-transducin repeat containing protein (β-TRCP) has been reported as a major pathway to degrade REST [12]. Additionally, aberrant splicing of *REST* leading to the expression of REST4, a truncated form of REST, has been reported in breast cancer samples and in the hippocampus [5, 51]. This truncation led to aberrant expression of REST target genes due to the loss of the repressor domain located in the C-terminus of REST [5, 51]. When full length REST was restored in small cell lung carcinoma it was able to induce apoptosis [9]. Elucidation of the exact mechanism leading to loss of REST function in breast cancer requires further studies. Our previous work in uterine fibroids has shown REST to be mislocalized and degraded in the cytoplasm. This mislocalization leads to aberrant expression of REST target genes that contribute to the pathogenesis of uterine fibroids [14]. While further studies are needed to determine how REST is lost, we found aberrant expression of REST targets in breast cancer are contributing to more aggressive forms of the disease. Therefore, we consider REST as an attractive target for therapy in breast cancer and other oncogenic diseases.

Gene expression analysis using TCGA showed loss of REST affects the regulation of genes involved in hormone signaling and hormone sensitivity. When ER is present a subset of REST targets are upregulated (Additional files 5 and 8). ADAM12 is a putative REST target (unpublished data) and has been shown to be regulated by estrogen [52]. In addition, ADAM12 expression has been shown to be a prognostic marker in ER + breast cancer samples [53]. As a disintegrin and metalloproteinase, it plays a role in cell–cell interactions and dysregulation of this gene has been associated with cancer progression [45]. IGF1 is also upregulated and is downstream of REST and ERα. Upregulation can lead to an increase in intracellular signaling and activation of the PI3K-AKT pathway and cell proliferation [46, 54]. These genes contribute to tumor growth in the presence of sex steroid hormones. In ER- or TNBC we see upregulation of REST target genes that play roles in hormone independent signaling. REST target genes upregulated in ER- or TNBCs include MYC, LIF, and EGFR which play significant roles in cancer progression, cell proliferation and endocrine resistance. Additionally, angiogenic factors including VEGFa and MMP9, REST target genes associated with migration and invasion of breast cancer cells, were upregulated in ER- or TNBC tumors. Based on our results showing a significant number of ERα targets being altered in the absence of REST as well as out IPA analysis data showing activation of estrogen signaling, we hypothesize REST plays a role in signaling downstream of ER and shares regulation of some ER target genes. REST’s regulation of ER targets is significant and shows RESTless tumors may respond differently to hormone-based treatment options. In addition, the mechanism by which they acquire endocrine resistance may differ from that of tumors with REST. Furthermore, we have data in the uterus showing that REST can be influenced by estrogen through enhancer of Zeste homolog 2 (EZH2) (unpublished data). This regulation by estrogen may affect which subset of REST targets are being expressed in the absence and presence of hormone receptors.

Our findings from gene expression analysis by RNA sequencing after gene knockdown in MCF-7 cells show loss of REST leads to activation of estrogen signaling pathway even in the absence of added estrogen (Fig. 2). RESTless tumors may have altered estrogen signaling even in the absence of added estrogen. In addition, alterations in the ligand independent ER pathway are a major factor that contribute to endocrine resistance [55]. A potential mechanism for this altered signaling includes activation of the PI3K/AKT pathway, which contributes to cell proliferation and growth in the absence of estrogen [55]. Our work in uterine fibroids shows when REST is lost it leads to overexpression of targets that contribute to PI3K/AKT activation [14]. In addition, the effect of estrogen is also amplified significantly in MCF-7 cells after REST knockdown, indicating that the loss of REST sensitizes breast cancer cells to estrogen signaling (Additional file 8). This is significant because estrogen signaling is responsible for activation of target gene transcription in several reproductive and non-reproductive organs [16]. In normal breast development estrogen signaling is responsible for ductal elongation, but in ER + breast cancer it promotes cell proliferation and progression of the tumor [16]. Estrogen receptor signaling is a common treatment target in breast cancer [56]. Nearly 70% of breast cancers express estrogen receptor (ER), progesterone receptor (PR), or both and proliferate in the presence of estrogen. Treatment with antiestrogens, aromatase inhibitors, or tamoxifen have been used to directly target the estrogen receptor or estrogen production and successfully reduced reoccurrence and improved mortality [16, 56]. Understanding how REST status affects estrogen signaling could help improve the efficacy of treatments for some patients. Although we do not know the mechanism that leads to the estrogen receptor signaling activation, our study shows RESTless tumors are hypersensitive to estrogen, may respond differently to hormone-based treatment and contribute to more aggressive forms of breast cancer.

We next wanted to determine the role of REST target genes that were contributing to the aggressive breast cancer phenotype. Our RNA sequencing data found *CEMIP* to be overexpressed in MCF-7 cells lacking REST. We confirmed CEMIP overexpression at both the mRNA and protein level in MCF-7 and MDA-MB-231 lacking *REST*. We also confirmed that high REST expression in other breast cancer cell lines correlates with low CEMIP expression (Fig. 4). However, CEMIP expression was found to be higher in the more aggressive cell line, MDA-MB-231 (Fig. 4). Overexpression of CEMIP has been previously reported in breast cancer patient samples and more aggressive breast cancer cell lines [17]. CEMIP expression is controlled through genetic and epigenetic mechanisms [49]. A study looking at truncated promoters of *CEMIP* showed AP-1 and NF-kB sites affect activity of the gene. However, DNA methylation status of a CpG island located within the first intron (+ 525 to + 1059) had the biggest impact on gene expression [49]. Hypermethylation of this region is seen in low expressing CEMIP breast cancer cell lines whereas hypomethylation is seen in high expressing CEMIP cell lines [49]. REST is known to silence its target genes through epigenetic mechanisms [7]. Studies show REST binds co-repressor mSin3 and forms a complex with histone deacetylases HDAC1 and HDAC2 and nuclear hormone receptor co-repressor (N-CoR) [57]. Furthermore, co-REST can bind REST and recruit methyl CpG binding protein (MeCP-2) and histone H3-lysine 9 methyltransferase G9a [58]. These unique complexes can alter the degree of gene repression by REST by changing epigenetic markers [59]. Analysis of available ChIP-Seq datasets (Additional file 9) showed RE1 sites present in the CEMIP gene. Interestingly, our study shows an RE1 site (+ 1022) located within the CpG island (+ 525 to + 1059). Using semi-quantitative ChIP-PCR we found that REST binds to this region and in the absence of REST this region may be influenced by other epigenetic changes like histone modifiers and transcriptional activators (Fig. 5). Future experiments using ChIP-qPCR are needed to confirm these modifications. Our current findings show REST binds to CEMIP locus and loss of REST leads to overexpression of CEMIP. It is known that histone H3K27 methylation is associated with cytosine methylation in CpG islands [60]. Further studies are needed to examine whether the loss of REST leads to altered CpG methylation of CEMIP regulatory regions within intron 1.

**Fig. 7:**
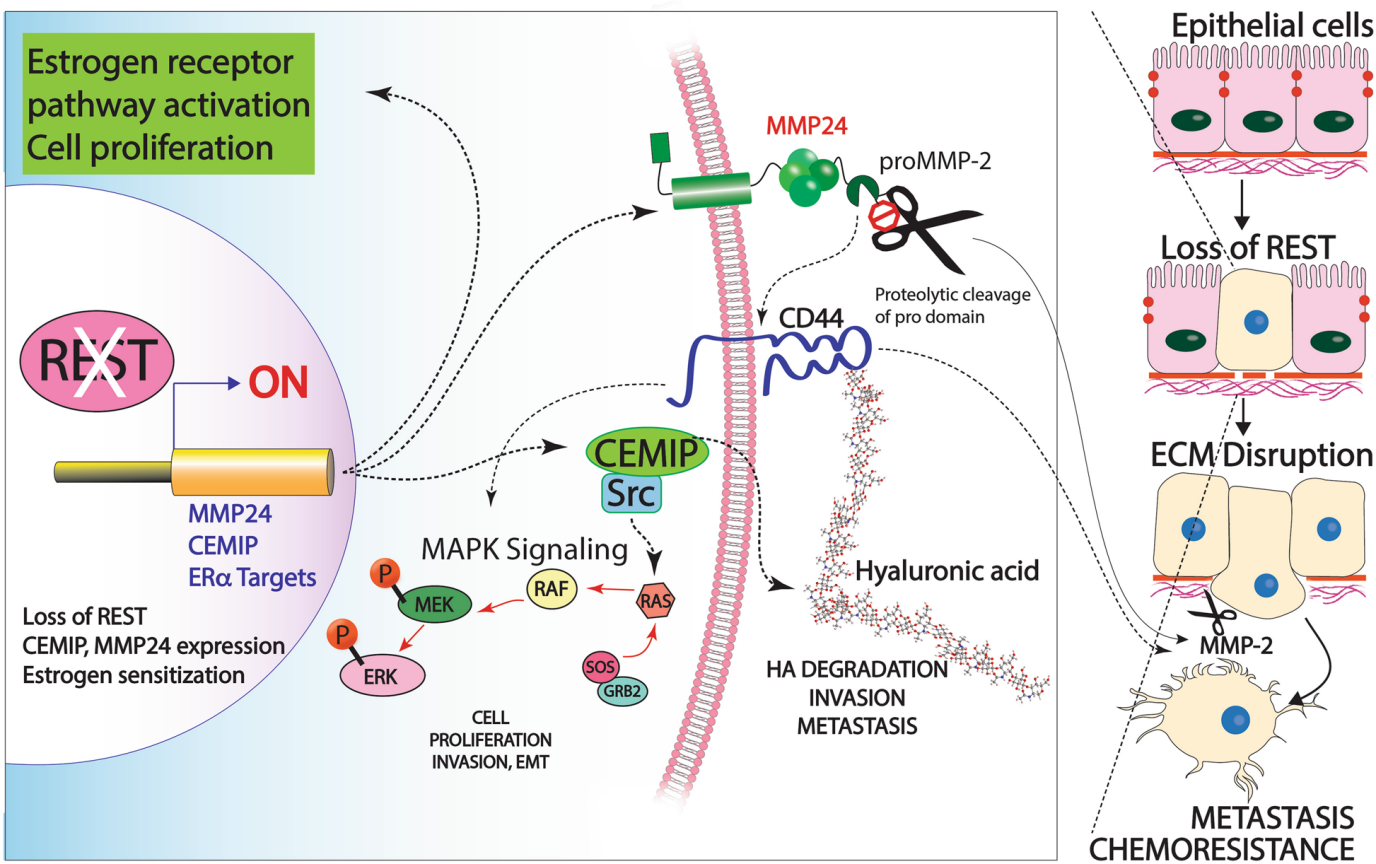
Loss of REST promotes estrogen sensitization, MMP24, and CEMIP overexpression leading to tumor progression and metastasis

Overexpression of CEMIP contributes to metastasis and invasion in RESTless breast cancer. Previous studies show CEMIP plays a critical role in hyaluronan (HA) depolymerization [26]. HA is a major component in the ECM and has a high turnover in physiological tissues. In healthy tissues, HA is found at a high molecular weight and provides structural integrity for the cells by binding its receptor, CD44 [61]. However, smaller fragments of HA are upregulated in cancer and promote ECM disruption, angiogenesis, invasion and metastasis [26]. Additionally, CEMIP has also been shown to activate intracellular signaling leading to cell survival and EMT [17, 23, 27]. In gastric cancer, CEMIP was shown to alter the expression of MMP2, a known player in EMT progression [62]. We show for the first time REST directly regulates *CEMIP* and loss of REST contributes to HA depolymerization and tumor progression by upregulating CEMIP (Fig. 7).

In addition to CEMIP, MMP24 is contributing to metastasis and ECM remodeling in RESTless tumors. MMP24 was predicted by ingenuity pathway analysis to have a role in cell–cell interactions, cell invasion, and extracellular matrix degradation. MMP24 is a membrane type matrix metalloproteinase that belongs to a family of zinc dependent endopeptidases [63]. Expression of MMP24 is mainly found in the nervous system, hippocampus, and cerebellum where it has a proposed role in neural plasticity and axonal growth [64]. Lack of MMP24, in a mouse model of thermal pain, was shown to affect synapse architecture and inhibit sensory neurons from transmitting pain stimuli [65]. MMP24 is responsible for cleaving ECM proteins, CD44, and MMP2 [32, 63, 66]. While the exact role of MMP24 in cancer is not clear, many studies show high expression is associated with poor outcomes [30, 63]. Wagoner et al. found that MMP24 is a part of a gene signature showing loss of REST function in both breast cancer patient samples and cell lines [5]. However, the mechanism of *MMP24* regulation was not shown. Given that REST suppresses neuronal genes in nonneuronal tissues we hypothesized REST directly regulates *MMP24*. Our results show a significant overexpression of *MMP24* mRNA in both MCF-7 and MDA-MB-231 cells in the absence of REST (Fig. 4). Furthermore, results from chromatin immunoprecipitation data show RE1 sites located in the promoter region and in the first intron of *MMP24* (Additional file 10). Semi-quantitative ChIP-PCR confirmed REST binding to the RE1 site located within the first intron of *MMP24*. In the absence of REST, there was less REST binding (Fig. 6). Based on previous studies, it is likely REST is regulating MMP24 expression in breast cancer patient samples. For future work, a validated, robust MMP24 antibody is needed to test how MMP24 protein levels change in the absence of REST for human patient samples. Here we show loss of REST leads to MMP24 mRNA overexpression and hypothesize this overexpression leads to aberrant cleavage of CD44 and MMP2 (Fig. 7).

Cleavage of CD44 by MMP24 activates intracellular signaling pathways, including PI3K/AKT and the Ras-MAPK pathway [32]. These pathways are involved in cell proliferation, motility and promote cellular invasion [55]. Based on known functions, we predict cleavage of CD44 also leads to the release of HA and allows for depolymerization by CEMIP to occur. Smaller molecular weight fragments of HA then contribute to angiogenesis, invasion and metastasis [26]. Additionally, MMP2 will be activated due to the increase in MMP24 and CEMIP [62, 66]. MMP2 activation will lead to degradation of the ECM, EMT, and further promote tumor invasion, angiogenesis and metastasis (Fig. 7) [20]. However, additional studies are needed to determine the exact roles CEMIP and MMP24 overexpression play in breast cancer. Future directions for determining the functional roles CEMIP and MMP24 play in breast cancer pathogenesis and metastasis will include invasion and migration assays in the presence and absence of REST, CEMIP and MMP24.

## Conclusions

In summary, we show here loss of REST plays an important role in promoting aggressive breast cancer (Fig. 7). We find REST alters the estrogen signaling pathway in hormone responsive breast cancer cells. This activation of estrogen signaling could lead to increased tumor size and disease progression. Furthermore, REST target genes are aberrantly expressed and have key roles in disease pathogenesis. In addition, REST transcriptionally regulates genes with known roles in invasion and metastasis, CEMIP and MMP24, by binding to RE1 sites located within their first intron. Our findings that REST contributes to aggressive forms of breast cancer by regulating metastatic and invasive genes provides an opportunity to further study REST as a treatment target. Additionally, REST’s role in other metastatic cancers has yet to be explored.

## Supplementary Information

**Additional file 1. **

**Additional file 2. **

**Additional file 3. **

**Additional file 4. **

**Additional file 5. **

**Additional file 6. **

**Additional file 7. **

**Additional file 8. **

**Additional file 9. **

**Additional file 10. **

**Additional file 11.**

## Data Availability

The datasets generated and analyzed during the current study are available in the GEO repository, GSE173857 or found in the additional files. To review GEO accession GSE173857: Go to https://www.ncbi.nlm.nih.gov/geo/query/acc.cgi?acc=GSE173857 Enter token ohmneqeofvklxur into the box.

## Abbreviations

RE1: Repressor element 1
ECM: Extracellular matrix
EMT: Epithelial-mesenchymal transition
ER: Estrogen receptor
PR: Progesterone receptor
TCGA: The Cancer Genome Atlas
IPA: Ingenuity pathway analysis
IDC: Invasive ductal carcinoma
DCIS: Ductal carcinoma in situ
TNBC: Triple negative breast cancer
HA: Hyaluronan
