# Supplementary material for: Loss of REST in breast cancer promotes tumor progression through estrogen sensitization, MMP24 and CEMIP overexpression
Source: BMC Cancer. 2022 Feb 17;22:180. doi: 10.1186/s12885-022-09280-2 (PMC8851790; doi:10.1186/s12885-022-09280-2)
Supplement: Supplementary file 1 — Additional file 1. [file 12885_2022_9280_MOESM1_ESM.docx]

**Additional file 1:**

REST ChIP-sequencing datasets

| **ENCODE Experiments** |
| --- |
| \| ENCFF986RRJ \| \| --- \| \| ENCFF896RCP \| \| ENCFF796YFZ \| \| ENCFF779CWH \| \| ENCFF713ZPE \| \| ENCFF706DRE \| \| ENCFF669XCW \| \| ENCFF668YET \| \| ENCFF540FXB \| \| ENCFF403CAJ \| \| ENCFF313CII \| \| ENCFF290ESJ \| \| ENCFF274BBE \| \| ENCFF208NUB \| \| ENCFF107EWI \| \| ENCFF023ZUW \| |
