# Supplementary material for: Loss of REST in breast cancer promotes tumor progression through estrogen sensitization, MMP24 and CEMIP overexpression
Source: BMC Cancer. 2022 Feb 17;22:180. doi: 10.1186/s12885-022-09280-2 (PMC8851790; doi:10.1186/s12885-022-09280-2)
Supplement: Supplementary file 2 — Additional file 2. [file 12885_2022_9280_MOESM2_ESM.docx]

**Additional file 2:**

Primers used for RT-qPCR assays

| **Gene Name** | **Assay ID** | **Company** |
| --- | --- | --- |
| 18S | Hs99999901_s1 | ThermoFisher |
| REST | Hs.PT.58.24545780 | Integrated DNA Technologies (IDT) |
| MMP24 | Hs00198580_m1 | ThermoFisher |
| MMP24 | Hs.PT.58.45636271 | Integrated DNA Technologies (IDT) |
| SNAP25 | Hs00938957_m1 | ThermoFisher |
| ESR1 | Hs01046816_m1 | ThermoFisher |
| PGR | Hs01556702_m1 | ThermoFisher |
| AP3B2 | Hs00190407_m1 | ThermoFisher |
| DISP2 | Hs00394338_m1 | ThermoFisher |
| BSN | Hs01109152_m1 | ThermoFisher |
| SYP | Hs00300531_m1 | ThermoFisher |
| FGF12 | Hs00912823_ m1 | ThermoFisher |
| HCST | Hs00367159_m1 | ThermoFisher |
| GABBR2 | Hs01554996_m1 | ThermoFisher |
| CHGA | Hs00900370_m1 | ThermoFisher |
| CPLX1 | Hs00362510_m1 | ThermoFisher |
| STMN3 | Hs00274822_m1 | ThermoFisher |
| CEMIP | Hs.PT.58.28305095 | Integrated DNA Technologies (IDT) |
