# Supplementary material for: Loss of REST in breast cancer promotes tumor progression through estrogen sensitization, MMP24 and CEMIP overexpression
Source: BMC Cancer. 2022 Feb 17;22:180. doi: 10.1186/s12885-022-09280-2 (PMC8851790; doi:10.1186/s12885-022-09280-2)
Supplement: Supplementary file 3 — Additional file 3. [file 12885_2022_9280_MOESM3_ESM.docx]

**Additional file 3:**

Sequences of primers used for ChIP-PCR assays

| **Primer Name** | **Sequence (5’-3’)** |
| --- | --- |
| MMP24 Site 1 Fwd | GGGGAAGAGGCTAAATCAG |
| MMP24 Site 1 Rev | GGAGTCACCCTCTCAGAC |
| MMP24 Site 2 Fwd | GCTGTCAGGTGGAGGTAG |
| MMP24 Site 2 Rev | CCAGTGATGAGCAGCTC |
| MMP24 Site 3 Fwd | GCCTAGCCCTATAGTATGG |
| MMP24 Site 3 Rev | GGCACCTTGTTATACCTC |
| CEMIP Site 1 Fwd 1 | GTGGGTCTGGTTTCTGG |
| CEMIP Site 1 Rev | GTCAGGATTCTCAGCCC |
| CEMIP Site 2 Fwd 3 | GACTGAGACTCCAAACG |
| CEMIP Site 2 Rev | GAATGTCACTGGCCTTC |
| CEMIP Site 3 Fwd 2 | GAGCGCGTCTCTCCATC |
| CEMIP Site 3 Rev | CAGCGGACTCTCAGTC |
