# Supplementary material for: Loss of REST in breast cancer promotes tumor progression through estrogen sensitization, MMP24 and CEMIP overexpression
Source: BMC Cancer. 2022 Feb 17;22:180. doi: 10.1186/s12885-022-09280-2 (PMC8851790; doi:10.1186/s12885-022-09280-2)

**Additional file 4:**

REST target gene expression in cancer. PLK1, ADAM12, TNNT1, and CEMIP (KIAA1199) are differentially regulated in many cancers. Using FireBrowse these genes were identified from the TCGA database.

**
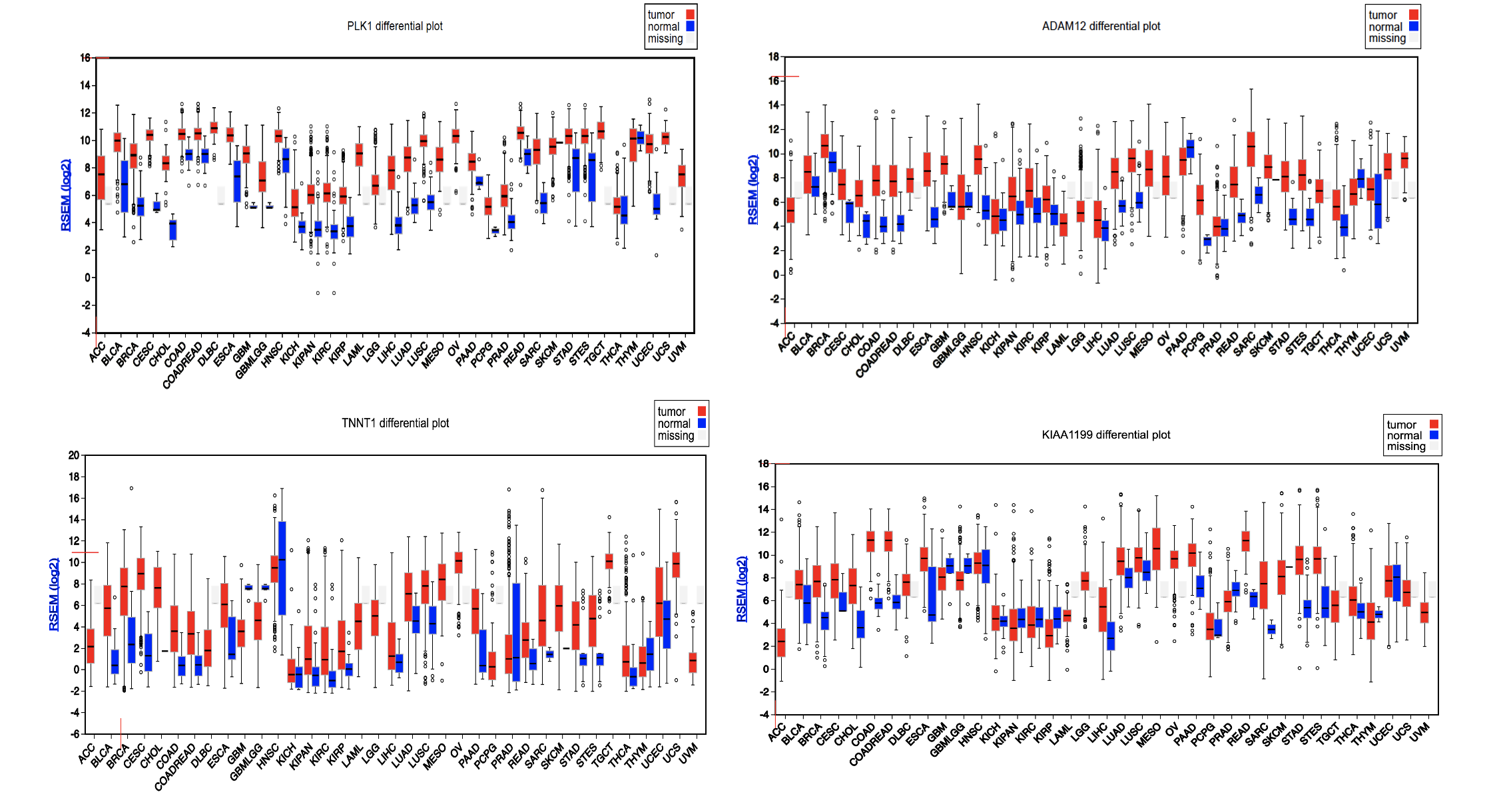
**


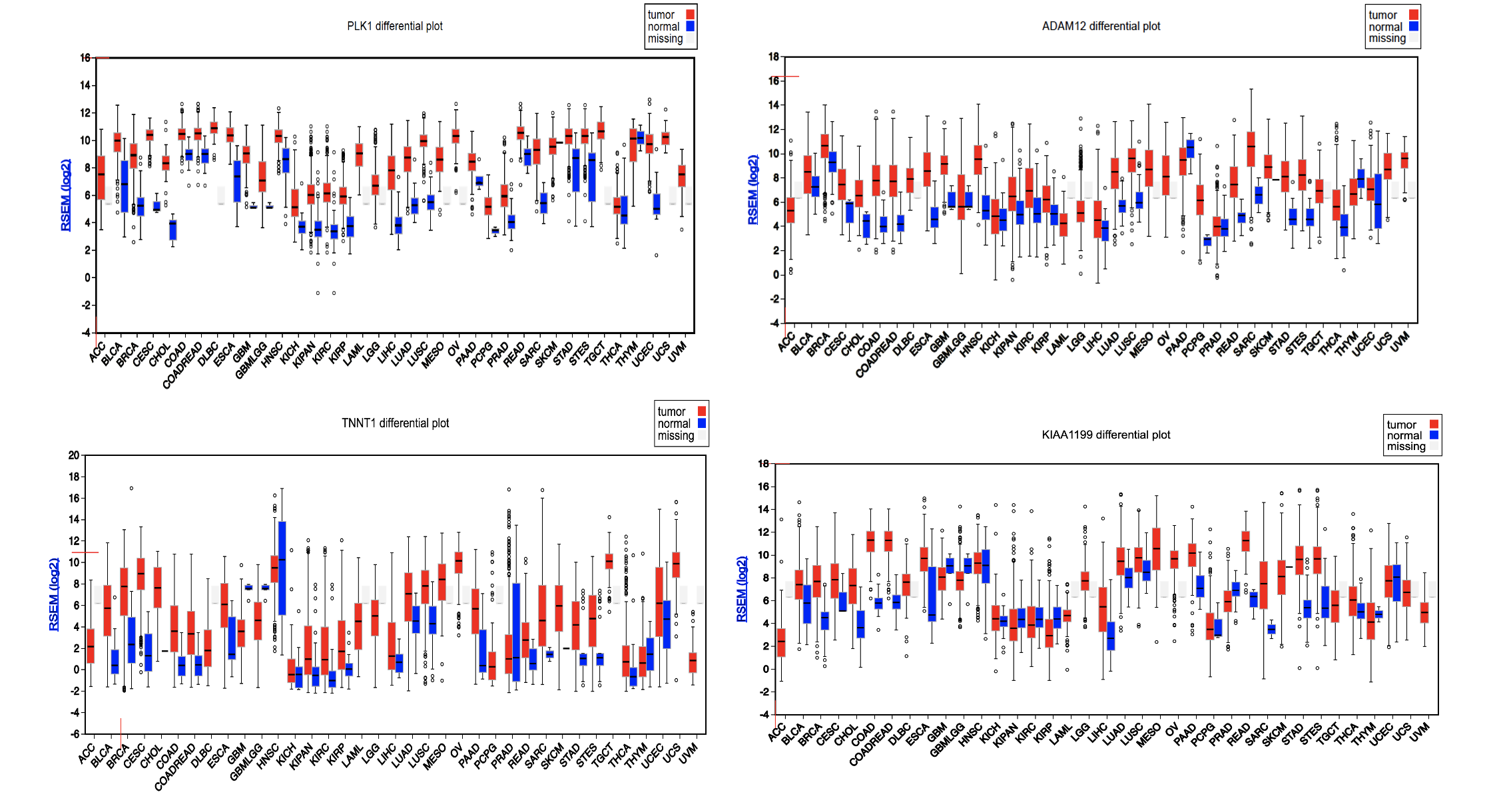

Supplement: Supplementary file 4 — Additional file 4. [file 12885_2022_9280_MOESM4_ESM.docx]
