# Supplementary material for: Loss of REST in breast cancer promotes tumor progression through estrogen sensitization, MMP24 and CEMIP overexpression
Source: BMC Cancer. 2022 Feb 17;22:180. doi: 10.1186/s12885-022-09280-2 (PMC8851790; doi:10.1186/s12885-022-09280-2)

**Additional file 5**

GEO dataset analysis. Hierarchical clustering of REST target downstream breast cancer gene expression data form A, GSE2034 and B, GSE19615.

A.


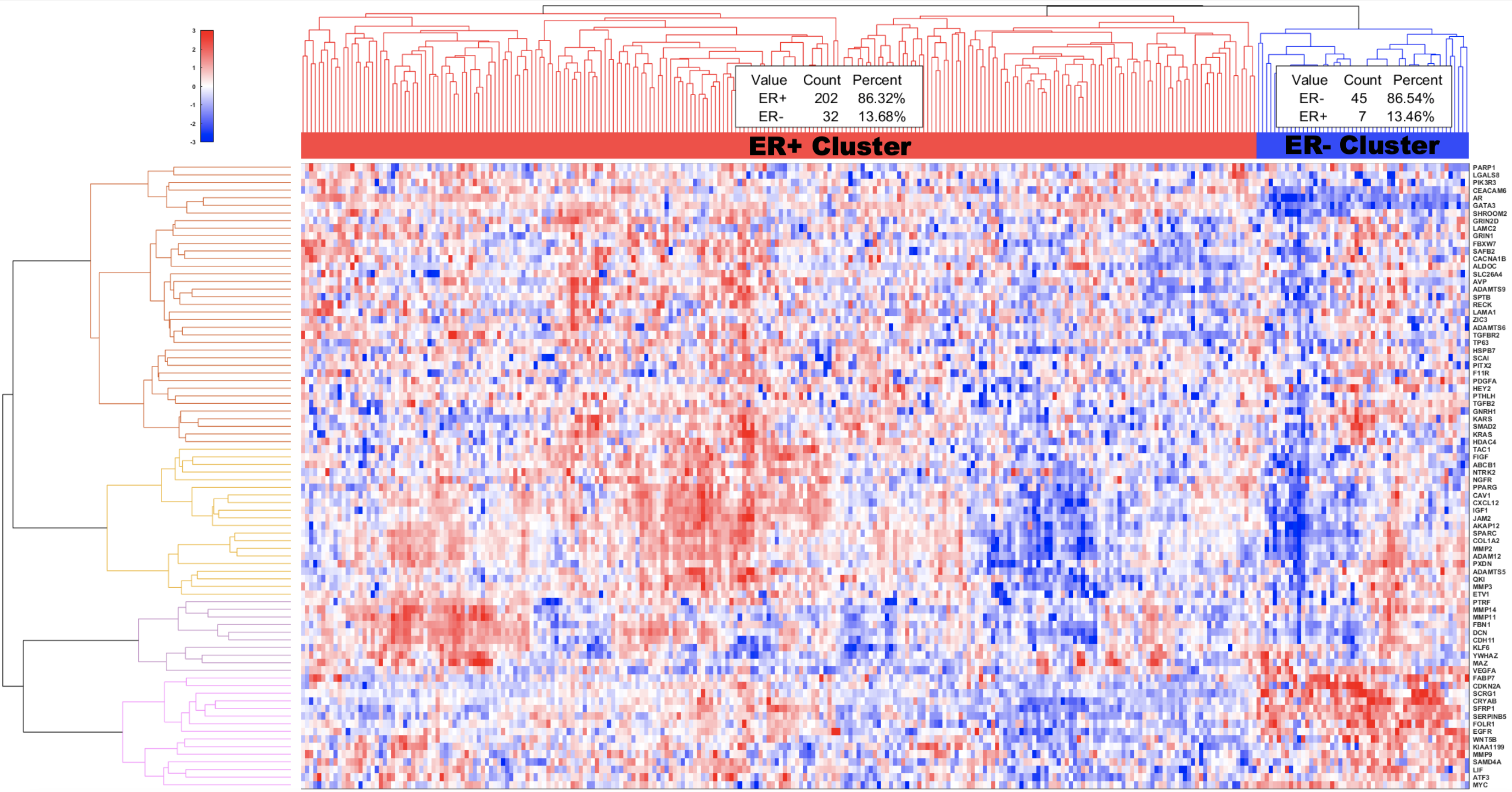


B.


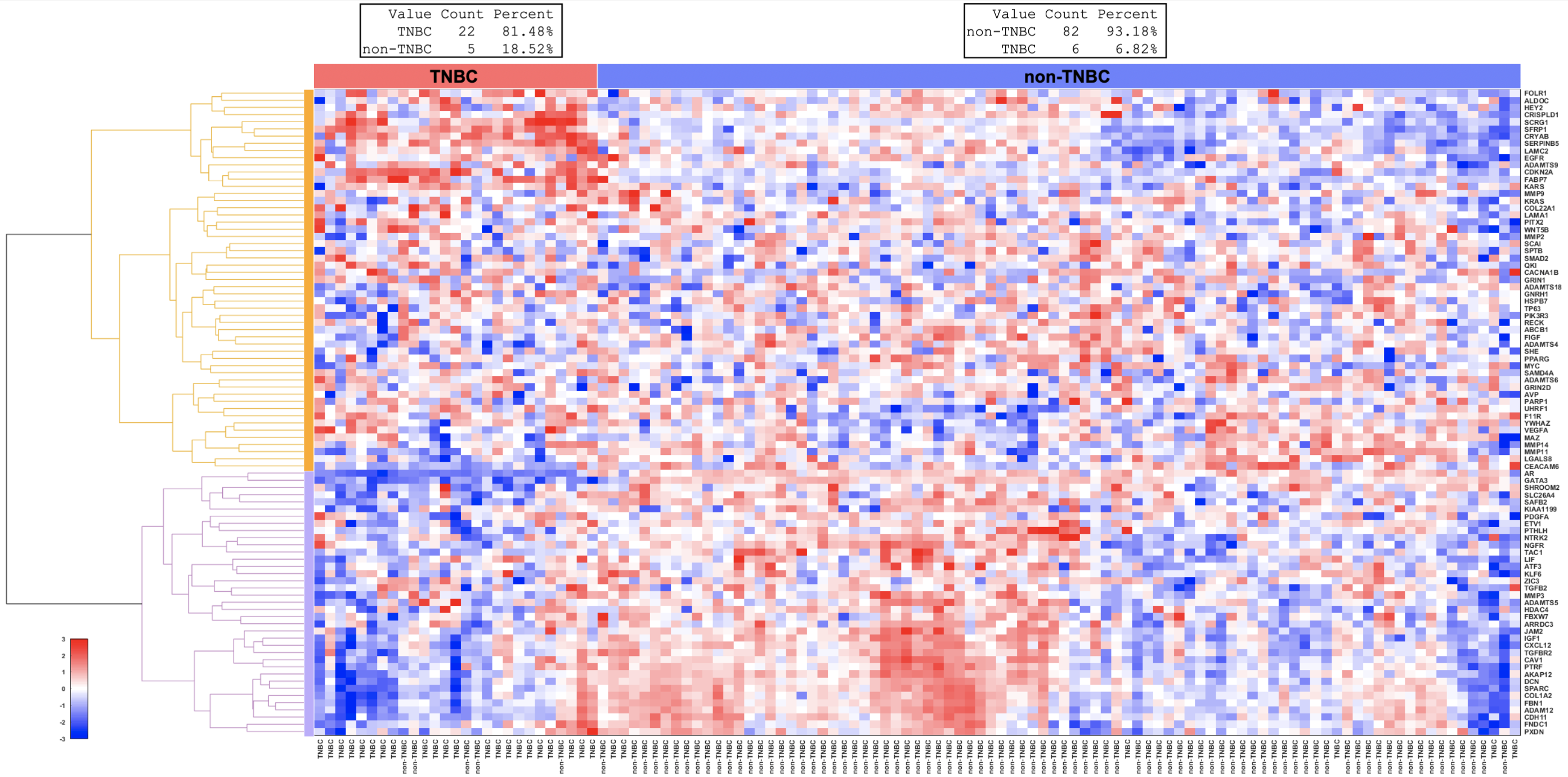

Supplement: Supplementary file 5 — Additional file 5. [file 12885_2022_9280_MOESM5_ESM.docx]
