# Supplementary material for: Loss of REST in breast cancer promotes tumor progression through estrogen sensitization, MMP24 and CEMIP overexpression
Source: BMC Cancer. 2022 Feb 17;22:180. doi: 10.1186/s12885-022-09280-2 (PMC8851790; doi:10.1186/s12885-022-09280-2)
Supplement: Supplementary file 6 — Additional file 6. [file 12885_2022_9280_MOESM6_ESM.docx]

**Additional file 6:**


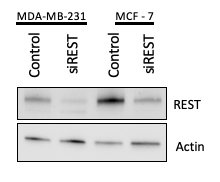


Endogenous REST expression in breast cancer cell lines. REST expression was knocked down in MDA-MB-231 and MCF-7 cell lines using lipofectamine delivery of siRNA targeting REST (siREST) compared to control.
