# Supplementary material for: Loss of REST in breast cancer promotes tumor progression through estrogen sensitization, MMP24 and CEMIP overexpression
Source: BMC Cancer. 2022 Feb 17;22:180. doi: 10.1186/s12885-022-09280-2 (PMC8851790; doi:10.1186/s12885-022-09280-2)
Supplement: Supplementary file 7 — Additional file 7. [file 12885_2022_9280_MOESM7_ESM.docx]

**Additional file 7**

Agglomerative hierarchical clustering of 1,219 significantly differentially expressed genes between treatment conditions.

Sample abbreviations: CV:Control, CP:Progesterone, CE:Estrogen, CEP:Estrogen & Progesterone, RV:REST knocked down, RP:REST knocked down & Progesterone, RE:REST knocked down & Estrogen, REP:REST knocked down & Estrogen & Progesterone
