# Supplementary material for: Loss of REST in breast cancer promotes tumor progression through estrogen sensitization, MMP24 and CEMIP overexpression
Source: BMC Cancer. 2022 Feb 17;22:180. doi: 10.1186/s12885-022-09280-2 (PMC8851790; doi:10.1186/s12885-022-09280-2)
Supplement: Supplementary file 8 — Additional file 8. [file 12885_2022_9280_MOESM8_ESM.docx]

**Additional file 8:**


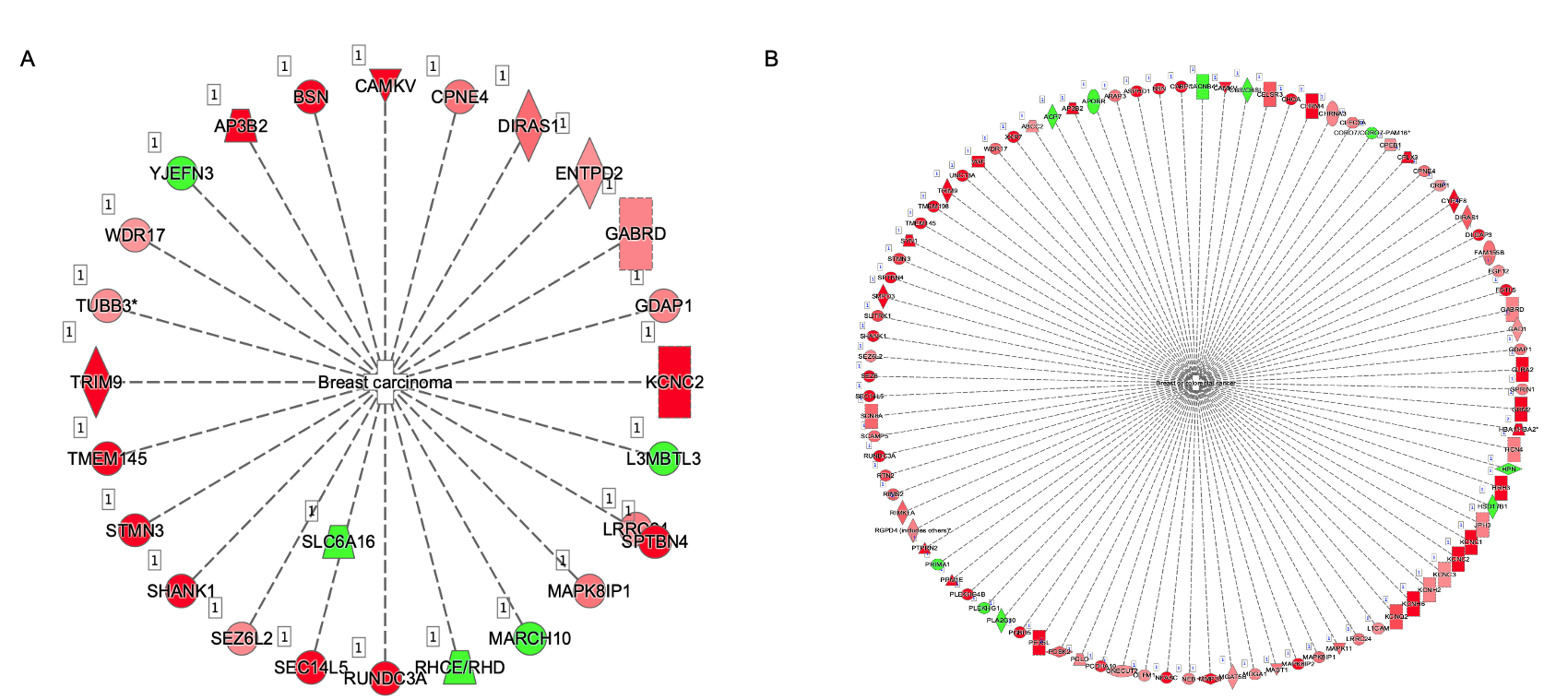


Analysis of breast cancer associated genes in MCF-7 *REST* knockdown cells. . A, Gene network analysis of breast carcinoma associated genes using Ingenuity Pathway Analysis software in MCF-7 cells transfected with siRNA for REST and treated with vehicle (PBS) compared to MCF-7 cells transfected with control siRNA and treated with vehicle (PBS). B, Gene network analysis of breast carcinoma associated genes using Ingenuity Pathway Analysis software in MCF-7 cells transfected with siRNA for REST and treated with estradiol (10nM) compared to MCF-7 cells transfected with control siRNA and treated with estradiol (10nM). Genes which were significantly (*p*<0.05) up-regulated (red) or down-regulated (green) in RNA-sequencing results.
