# Supplementary material for: Loss of REST in breast cancer promotes tumor progression through estrogen sensitization, MMP24 and CEMIP overexpression
Source: BMC Cancer. 2022 Feb 17;22:180. doi: 10.1186/s12885-022-09280-2 (PMC8851790; doi:10.1186/s12885-022-09280-2)
Supplement: Supplementary file 11 — Additional file 11. [file 12885_2022_9280_MOESM11_ESM.docx]

Additional file 11

Full western blots from Figure 2A

Top: REST


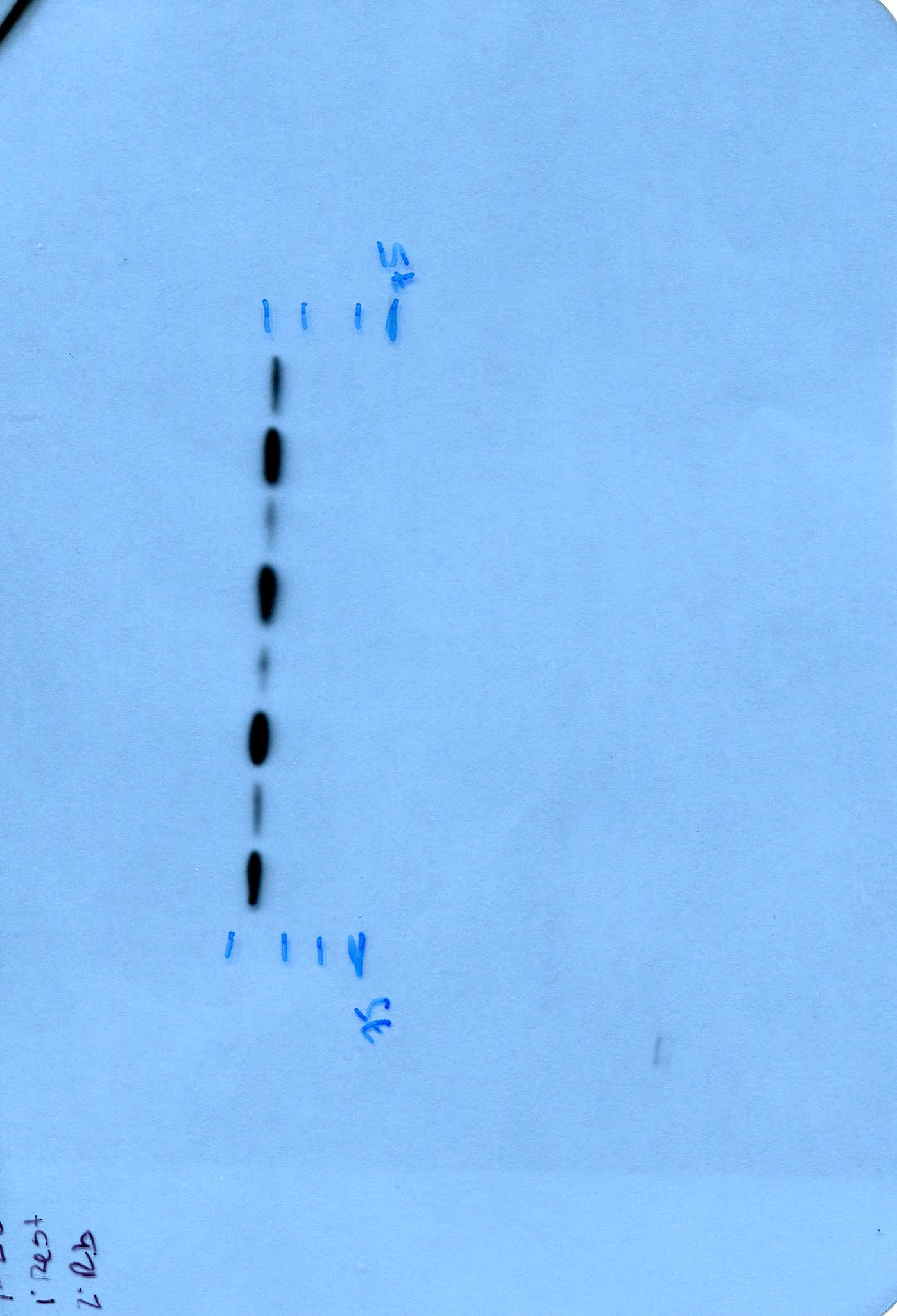


Bottom: Actin


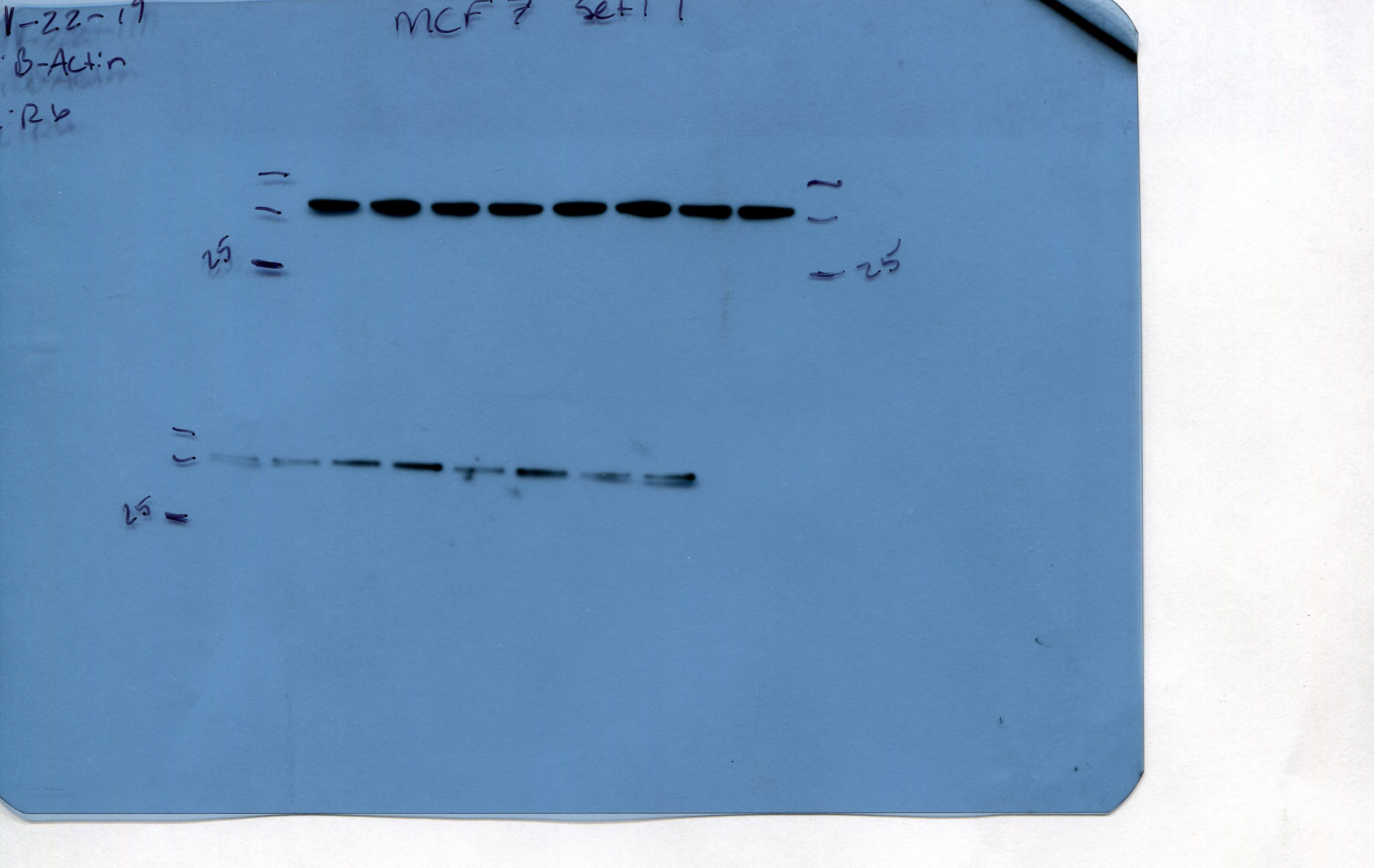


Full western blots from Figure 4A

Top: REST


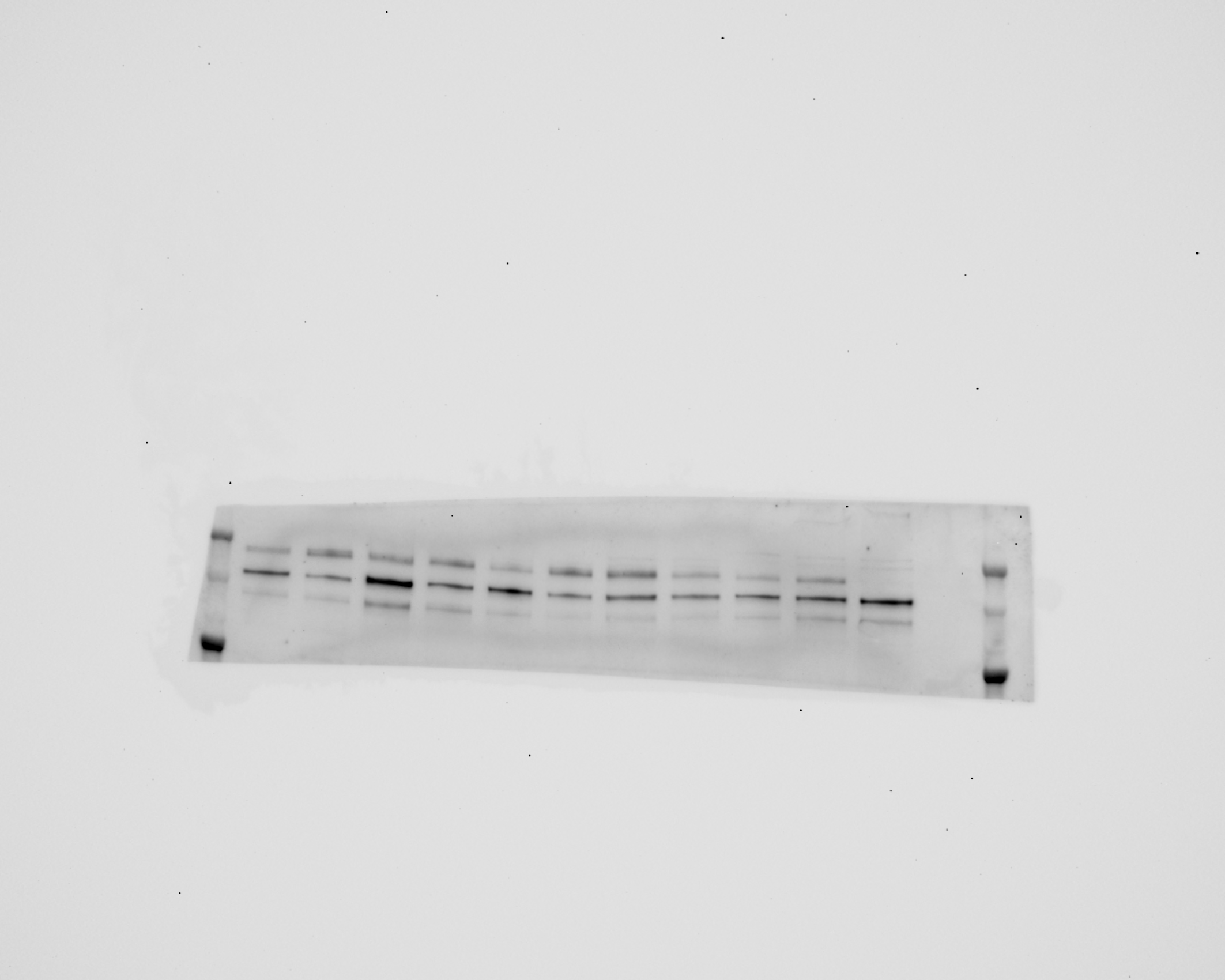


Middle: CEMIP


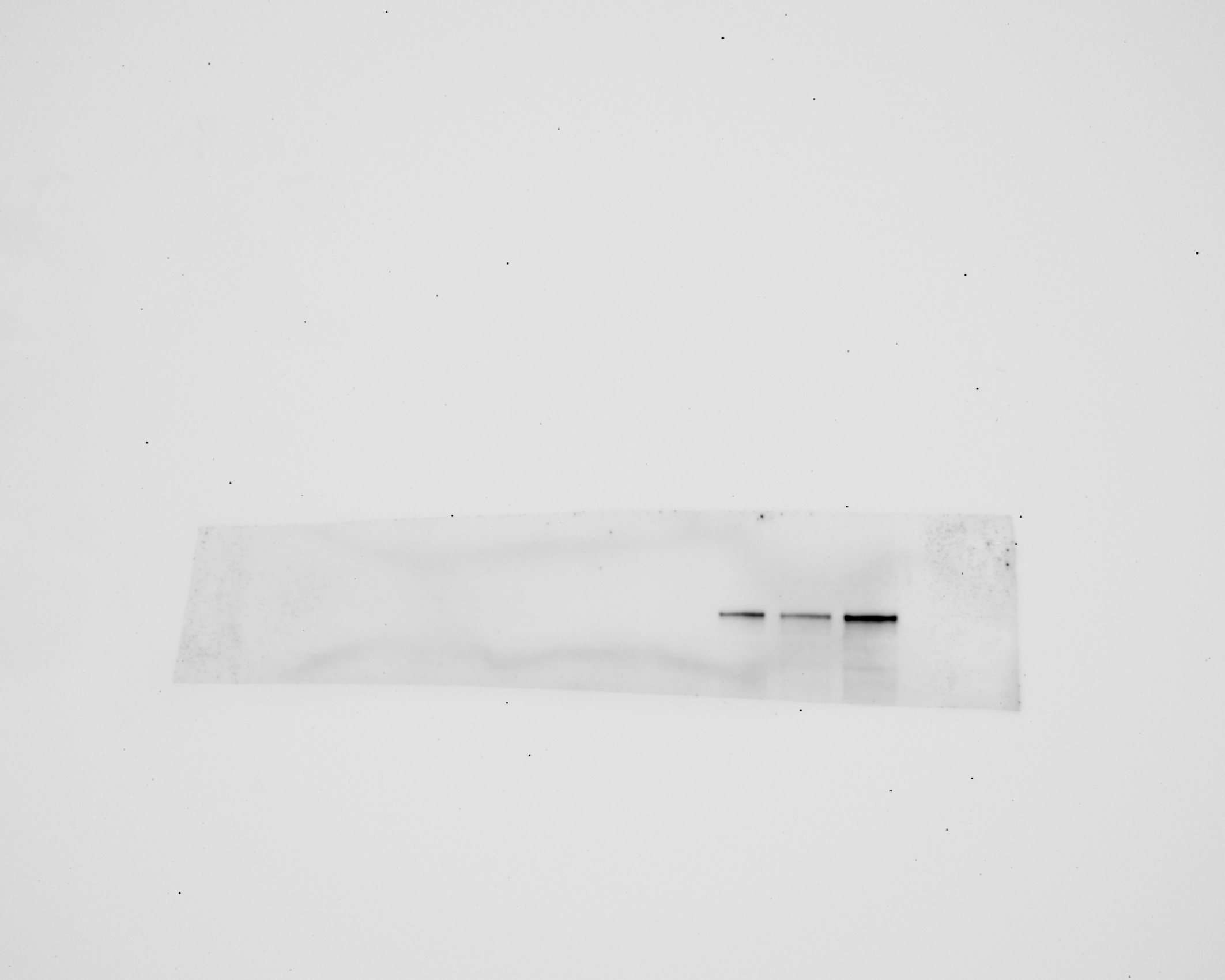


Bottom: B-actin


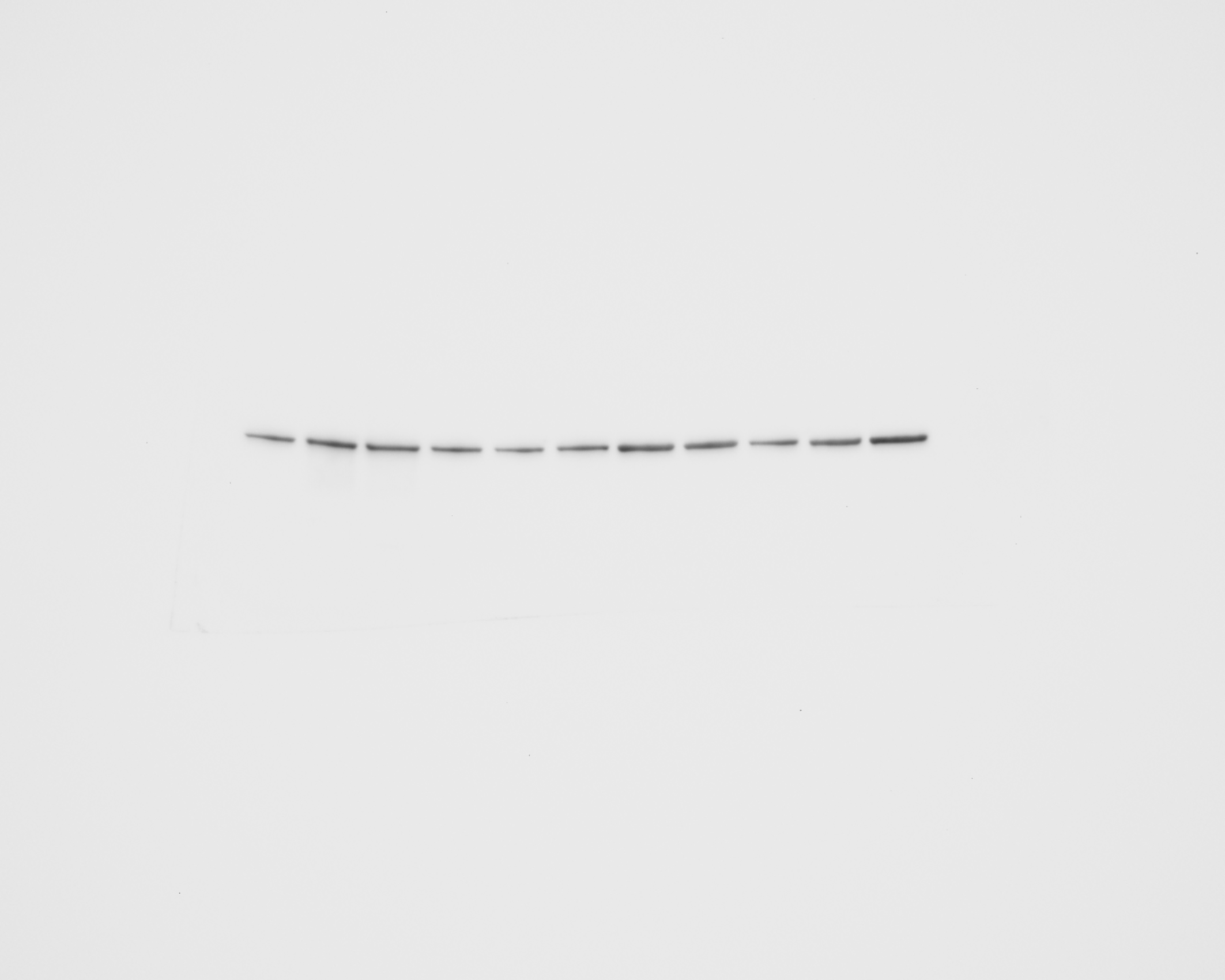


Full western blots from Figure 4C

Top: CEMIP


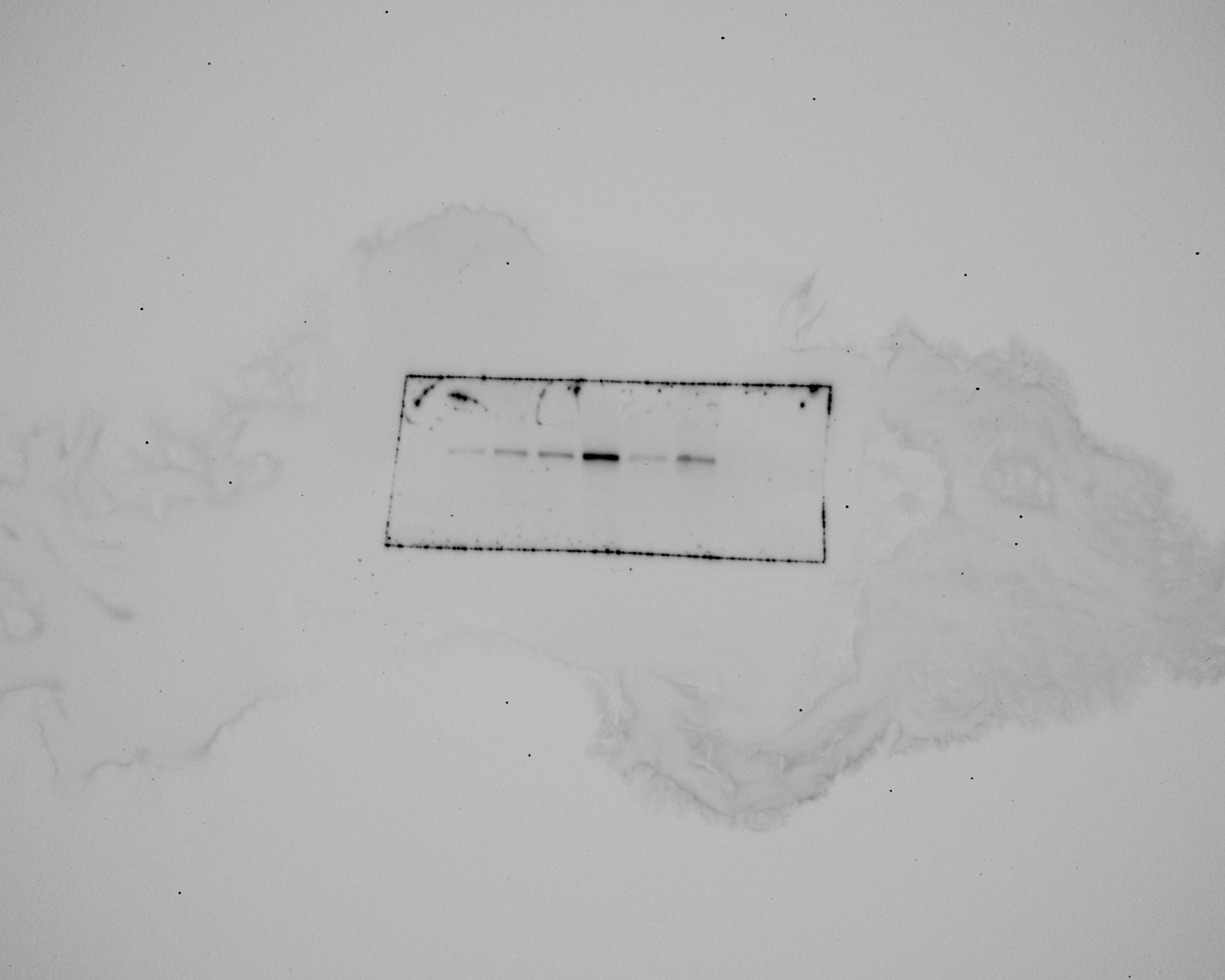


Middle: REST


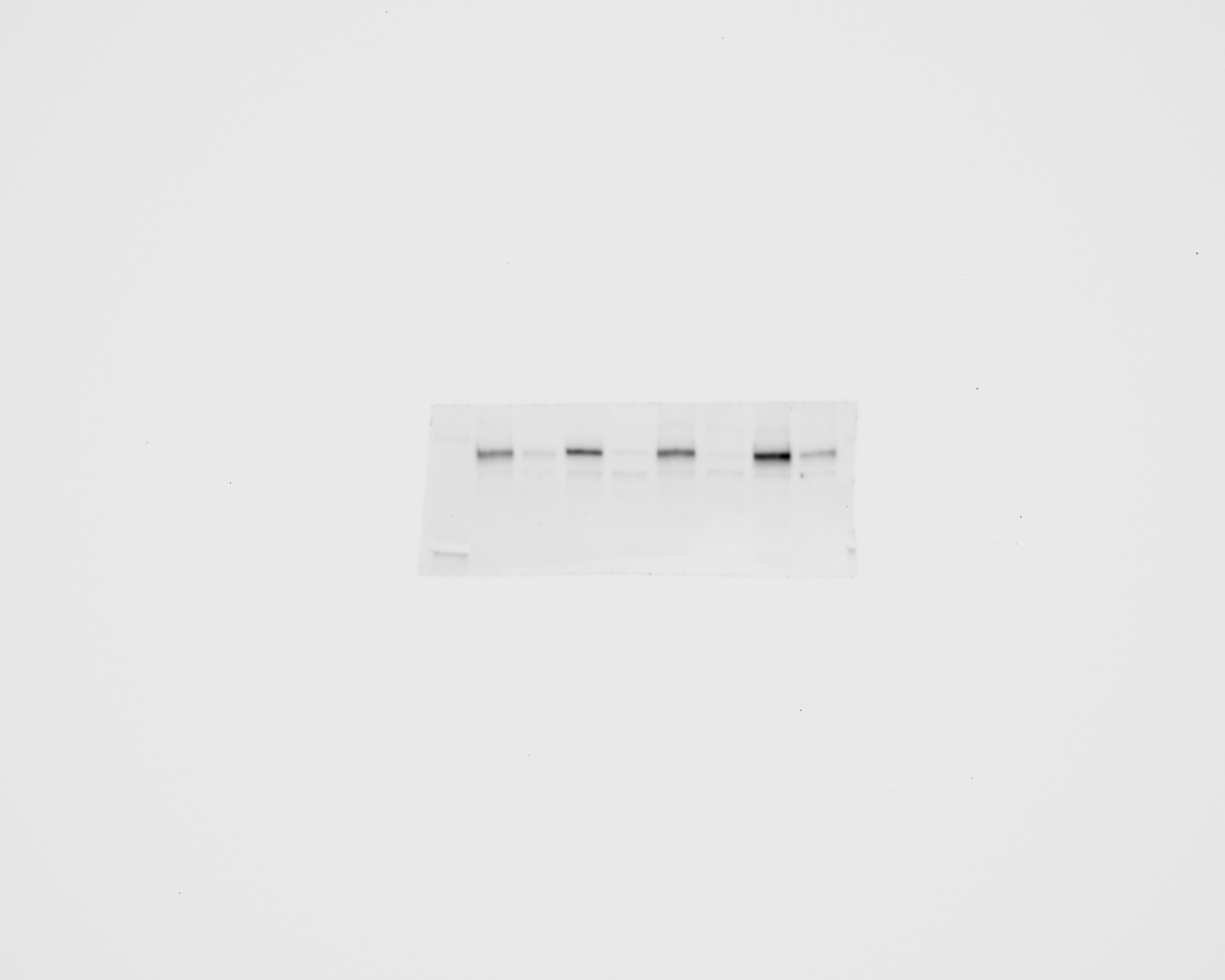


Bottom: Actin


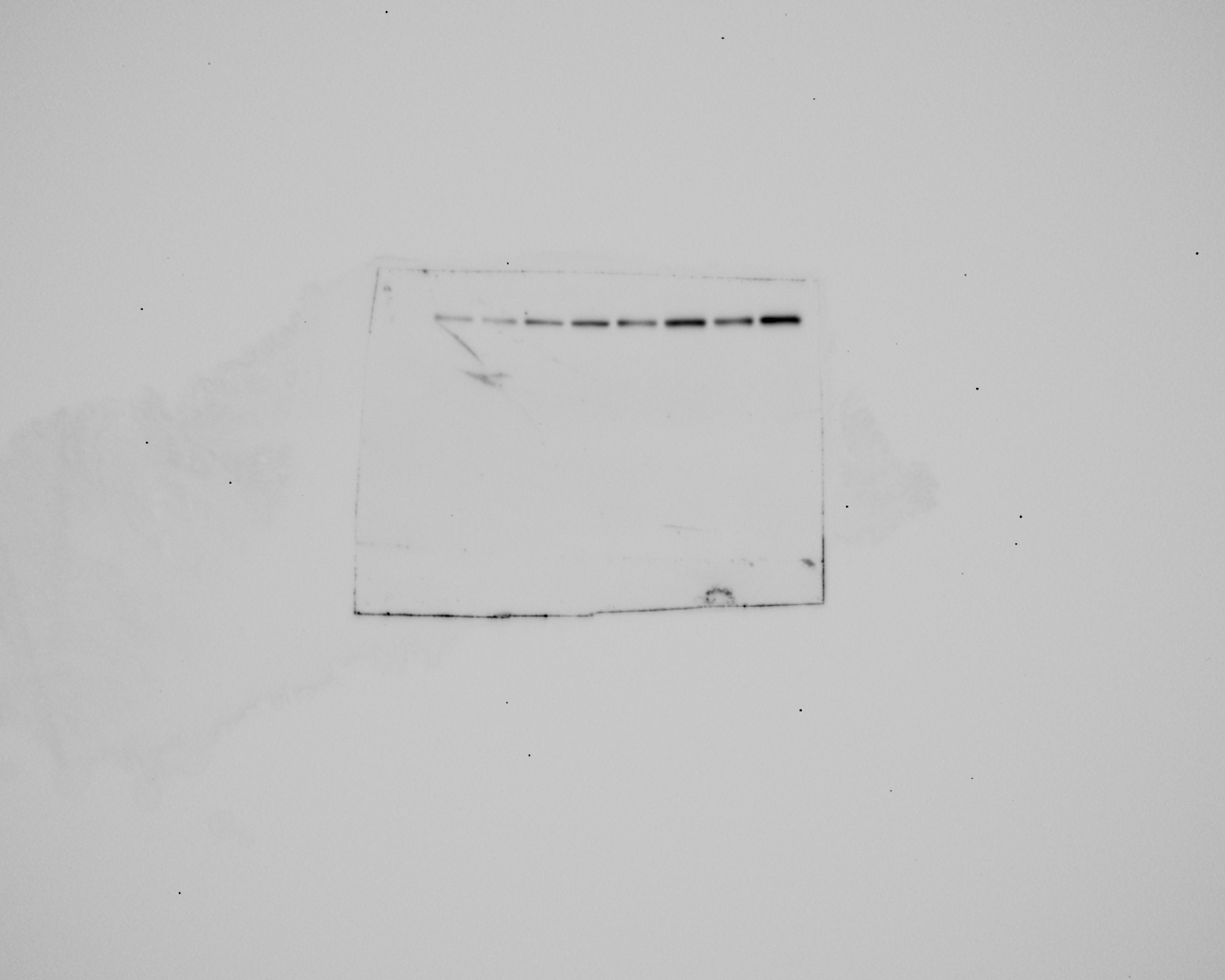


Full PCR Gels from Figure 5B

Site 1


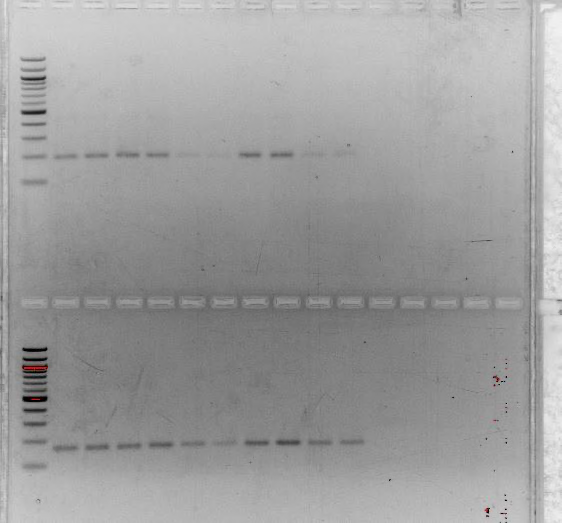


Site 2


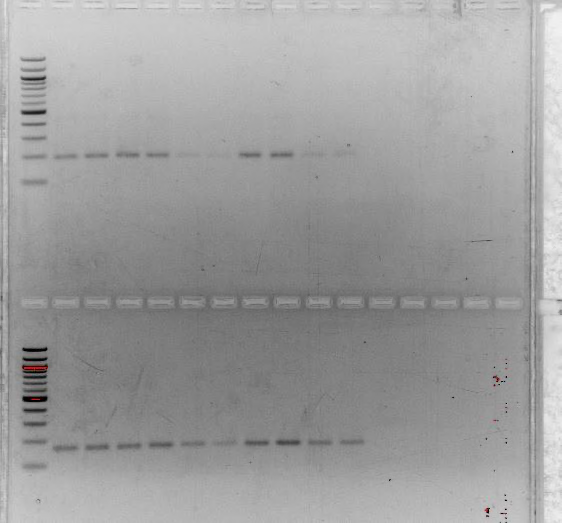


Site 3


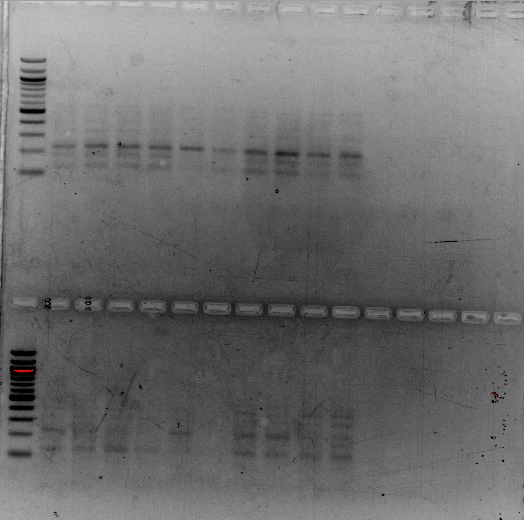


Full images of PCR gels from Figure 6B

Site 2


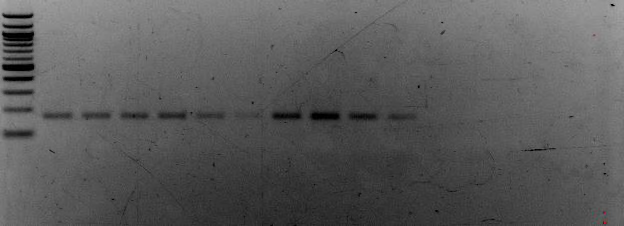


Site 3 top gel & Site 1 bottom gel


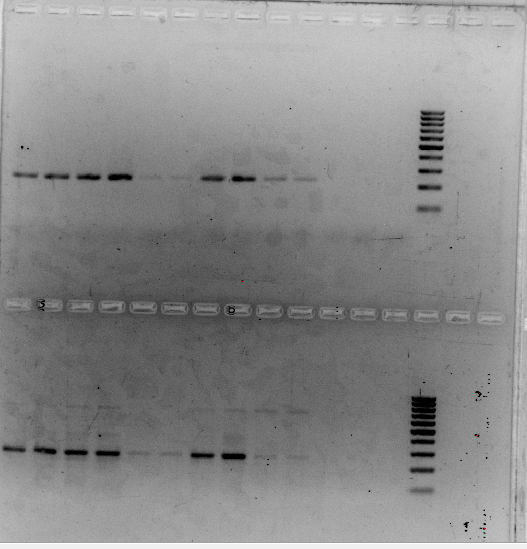


Full blots of REST and Actin from Additional file 6

Top:REST


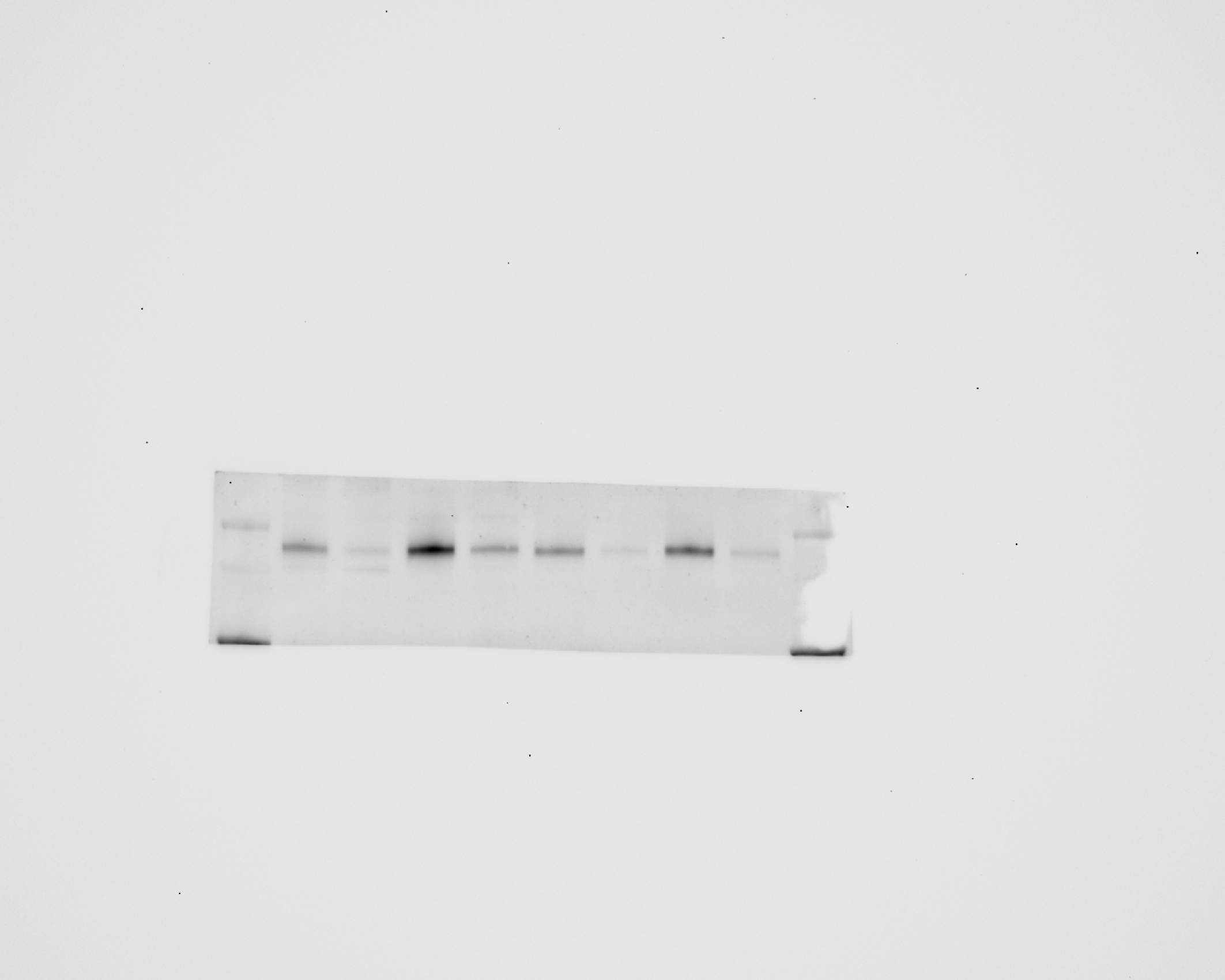


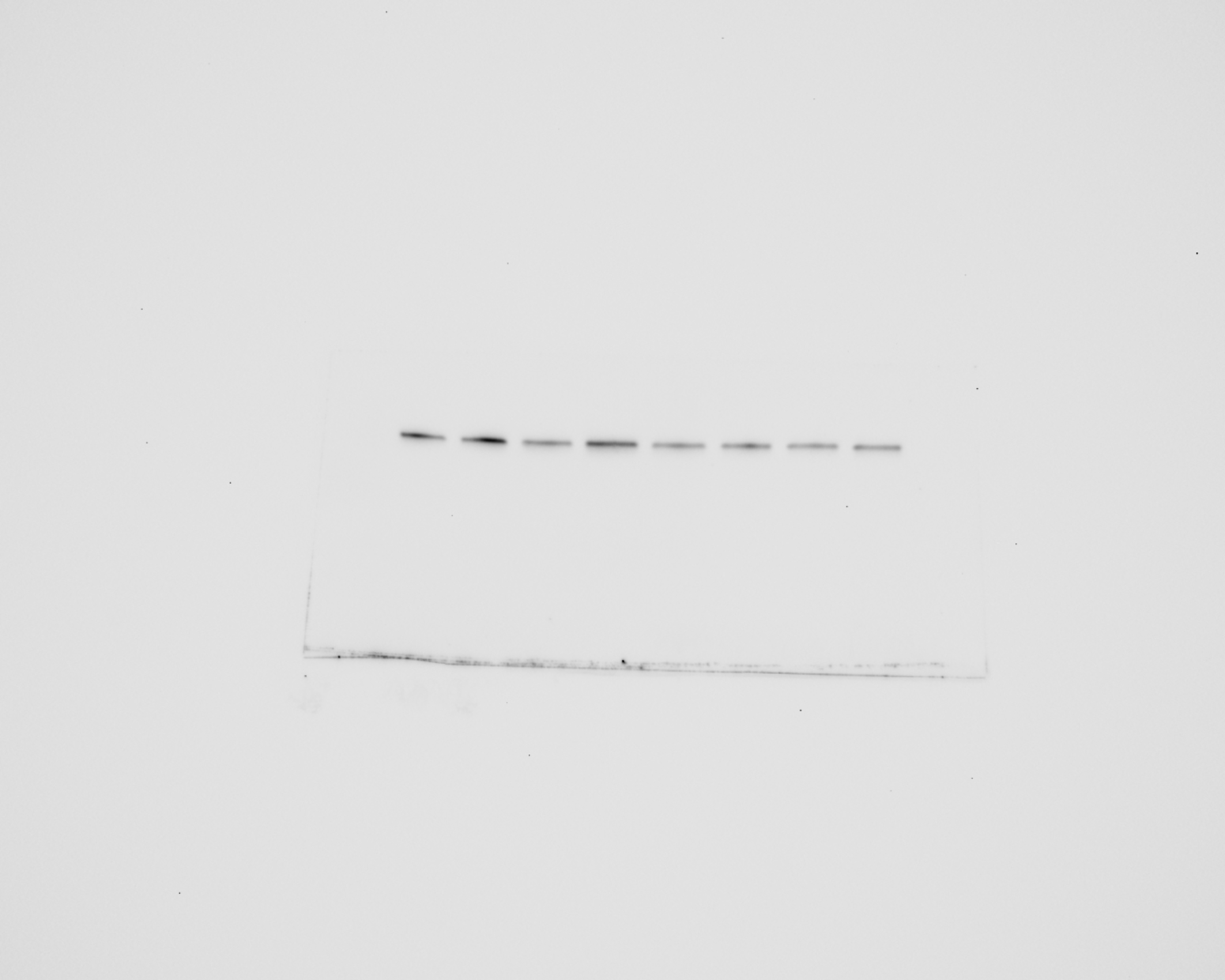


Bottom: B-actin
